# Distinct Interactions of Cannabinol and Its Cytochrome P450-Generated Metabolites with Receptors and Sensory Neurons

**DOI:** 10.1021/acs.jmedchem.5c00938

**Published:** 2025-06-26

**Authors:** Debanjan Kundu, Luca Franchini, Hale S. Hasdemir, Elliot Lloyd, Jonathan Maturano, Katalin Rabl, Anna Nicole Denissiouk, Mark Schumacher, David Sarlah, Judith Hellman, Emad Tajkhorshid, Cesare Orlandi, Aditi Das

**Affiliations:** † School of Chemistry and Biochemistry, College of Sciences, Georgia Institute of Technology, IBB, 1372 Parker H. Petit Institute for Bioengineering and Biosciences, Atlanta, Georgia 30332, United States; ‡ Department of Pharmacology and Physiology, 6923 University of Rochester Medical Center, 601 Elmwood Ave, Rochester, New York 14642, United States; § Theoretical and Computational Biophysics Group, NIH Resource for Macromolecular Modeling and Visualization, Beckman Institute for Advanced Science and Technology, Department of Biochemistry, and Center for Biophysics and Quantitative Biology, 14589 University of Illinois Urbana−Champaign, Urbana, Illinois 61801, United States; ∥ Roger Adams Laboratory, Department of Chemistry, Cancer Center at Illinois, University of Illinois, Urbana, Illinois 61801, United States; $ Department of Chemistry, 124473 Rice University, Houston, Texas 77005, United States; # Department of Anesthesia and Perioperative Care, 8785 University of California San Francisco, San Francisco, California 94143, United States

## Abstract

Interest in nonpsychotropic cannabinoids like cannabinol (CBN) is increasing for pain therapy. This study delivers critical insights into CBN’s metabolism and pharmacological effects, uncovering its therapeutic potential for pain reduction. Using metabolomics, we identify CBN-11-OH as the dominant metabolite, with lower levels of CBN-1′-OH and CBN-quinone. Computational simulations reveal CBN’s stability at the CYP2C9 active site, driving hydroxy metabolite formation. We report the intricate biotransformation of CBN by multiple cytochrome P450 enzymes. CBN and its metabolites exhibit mild anti-inflammatory effects in microglial cells, though less potent than cannabigerol and cannabichromene. Receptor activation assays further reveal that CBN-1′-OH acts as a partial CB1 agonist, while CBN and its metabolites antagonize CB1 and CB2 receptors. Notably, CBN and CBN-11-OH elevate intracellular Ca^2+^ levels in dorsal root ganglia sensory neurons—an effect linked to potential pain relief. These findings lay the groundwork for harnessing CBN and its metabolites in novel pain therapeutics.

## Graphical abstract

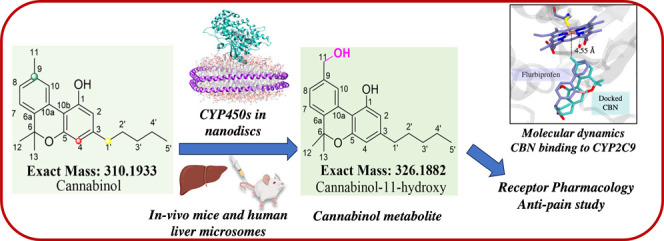

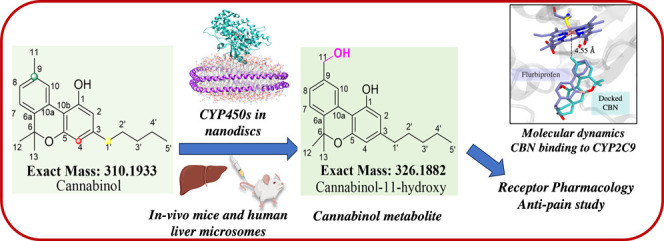

## Introduction

Cannabinol (CBN) is a phytocannabinoid that shares structural similarities with Δ9-tetrahydrocannabinol (Δ9-THC) but is devoid of potent psychoactive effects, which makes it a mild sedative, earning it the nickname “the sleep cannabinoid”. CBN is found in increased amounts in when the plant is aged and is the oxidative degradation product of THC.^1^ CBN has unique pharmacological properties. It has been reported to be utilized in the relief of chronic pain, like temporomandibular disorders and fibromyalgia, in rat models for myofascial pain.^2^ Compared to cannabidiol (CBD) and Δ9-THC, CBN has an antiallergic effect on airway infections by inhibiting the production of various interleukins and reducing mucus production in mouse models.^3,4^ Lastly, compared with other cannabinoids like cannabichromene (CBC) and cannabigerol (CBG), CBN is reported to be effective against methicillin-resistant (MRSA).^5^

Due to the growing use of CBN as a mild sedative and analgesic, it is essential to understand its metabolism by human cytochrome P450s, which are the phase I drug-metabolizing enzymes involved in the metabolism of most xenobiotics. Previously, we have shown that CYPs rapidly convert CBG and CBC to form cyclo-CBG and hydroxy CBC, which have a distinct pharmacology from the parent CBG and CBC themselves.^6,7^

Phytocannabinoids broadly target the endocannabinoid system of the body, which consists of cannabinoid receptors 1 and 2 and the endocannabinoids.^8,9^ CBN was shown to have lower binding affinities for both cannabinoid receptors CB1 and CB2 than Δ9-THC. The study reports CBN levels to act in the high nanomolar range (392.2 ± 53.5 nM) for CB1 receptors and inhibition of adenylyl cyclase in brain synapses. Furthermore, it reports the binding of CBN to CB1 and CB2 receptors in COS-7 cell lines, with *K*_i_ values of 211.2 ± 35.0 nM for CB1 and 126.4 ± 26.0 nM for CB2 receptors, and similar inhibition of adenylyl cyclase.^10^

CBN is also an agonist for various TRP channel receptors like TRPV4. A study examining TRP channel activation and desensitization measured the efficacy of CBN in activating TRPV4 compared to a full agonist. The study reported that CBN had low efficacy (15.3–26.1%) compared to ionomycin. The potency of carvacrol, measured in the reported study, is the cannabinoid’s potential to prevent an increase in intracellular calcium ions upon stimulation by 1 mM of carvacrol. CBN was reported to have an IC50 of 9.4 ± 0.1 μM as its measure of potency^11–13^

The major metabolic products of Δ9-THC are 11-OH-THC and THC–COOH in rat and rabbit liver preparations.^14,15^ Although CBN is a derived product of THC, structurally, CBN has an additional aromatic ring compared to THC (Figure 1), which confers additional stability to the molecule that slows down its rate of metabolism by CYP2C9 and CYP3A4. At the same time, interactions with other CYPs have not been studied.^16,17^ CBN has no double-bond isomers or stereoisomers.^16,18^ Previous literature suggests that the first-pass metabolism of CBN in the liver leads to the hydroxylation at the C11 position.^19,20^ CBN and its metabolites are also glucuronidated by Phase II UGT enzymes, specifically by UGT isoforms 1A7, 1A8, 1A9 and 1A10.^14^

**Figure 1:**
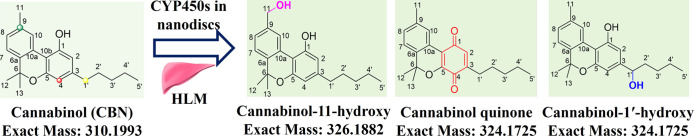
Structural representation of cannabinol (CBN) metabolism. Metabolism by purified CYPs leads to the formation of various metabolites like CBN-1′–OH, CBN-11-OH, and CBN-quinone (*p*-CBNQ).

In this current study, we have synthesized CBN and some of the major metabolites like CBN-1′–OH, CBN-11-OH and CBN-*p* quinone (Figure 1). We also developed a targeted mass spectrometry method to detect CBN, CBN-1′–OH, CBN-11-OH and CBN-quinone. We used this method to evaluate the formation of the major CBN metabolites using human liver microsomes (HLMs), purified CYPs (CYP3A4, CYP2D6 and CYP2C9) incorporated into nanodiscs and compared the metabolism profile with *in vivo* experiment of mice fed with CBN. We utilized HLMs to also identify potential glucuronidation products of CBN and CBN-11-OH using untargeted mass spectrometry. Furthermore, we used molecular dynamics (MD) to get insight into the molecular interactions of CBN and related cannabinoid THC with the active site of CYP2C9, which is the most common cytochrome P450 involved in cannabinoid metabolism. Additionally, we studied the in-depth activation of cannabinoid receptors by CBN and its metabolites. We studied the potency of CBN both as an agonist and antagonist at both cannabinoid receptor 1 (CB1R) and cannabinoid receptor 2 (CB2R), employing the interactions of CBN and its metabolites with microglial cells and neurons.

## Results

### Synthesis of CBN Metabolites

Commencing from commercially available CBN, CBN-quinone (*p*-CBNQ)^1^ (**1**) was accessed in 57% yield by utilizing SeO_2_ and TBHP (Figure 2) Accessing oxidation at the 1′ position required first acetylation to protect the phenol to afford CBN-Ac (**2**) in 85% yield. This material could then be heated to 50 °C with NBS and AIBN to undergo a Wohl–Ziegler bromination, accessing the 1′ benzylic bromide **3**. This crude material was directly subjected to AgOAc to perform acetoxylation to diacetate **4** in 32% yield across two transformations. Both acetates were deprotected with K_2_CO_3_ and MeOH, affording racemic 1′-hydroxy-CBN (**5**) in 70% yield. The molecules were characterized using NMR and mass spectrometry, as described in the Experimental Section under synthesis of CBN-based compounds.

**Figure 2:**
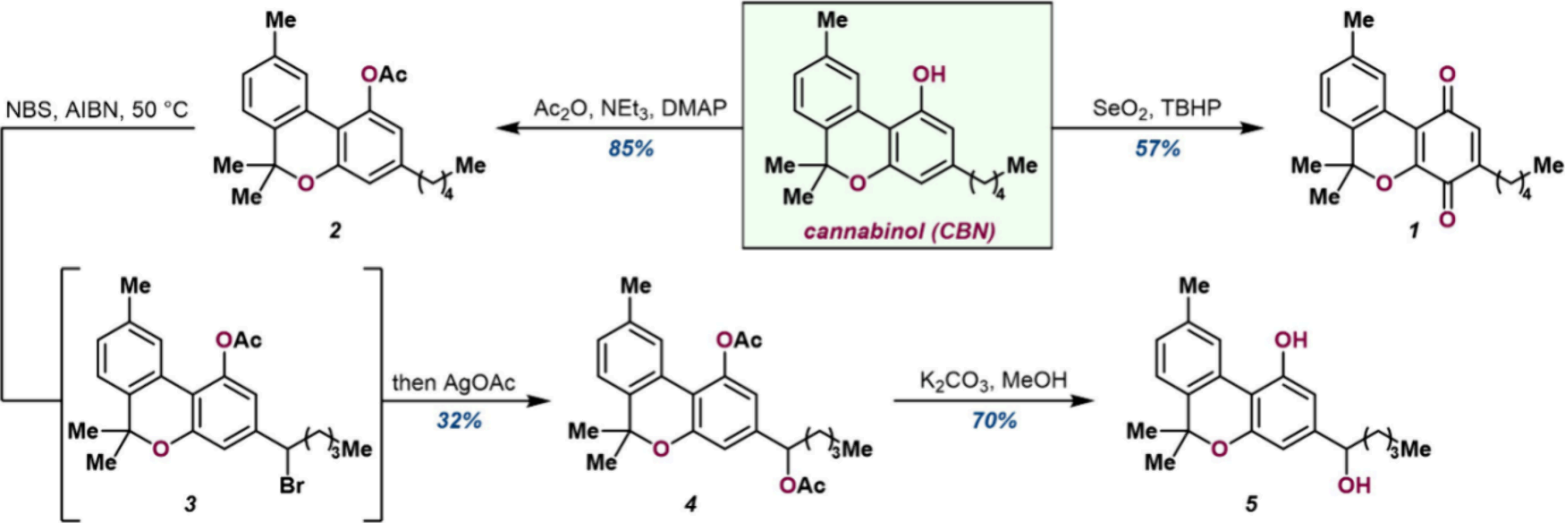
Synthesis scheme of various cannabinol metabolites. Methods are available in the Experimental Section below (SI Sections 1(1.1–1.3)).

#### Direct Metabolism of Cannabinol (CBN) and Secondary Metabolism of CBN Metabolites by Human Liver Microsomes Determined Using LC-MS/MS

We report the direct metabolism of CBN by cytochrome P450s and identify its metabolites using LC-MS/MS. The primary metabolite was identified as CBN-11-OH, along with other metabolites, including CBN-1′–OH and CBN-quinone. Using LC-MS/MS, we identified the peak of the parent CBN molecule eluting at 7.84 min. The mass fragmentation patterns of CBN (Figure 3A,B) are identical to the previously reported pattern.^21^ The primary metabolism products of CBN included the CBN-11-OH (Figure 3C,D) and CBN-1′–OH. We also detected the formation of CBN-11-OH from the metabolism of CBN by purified CYP2C9 and CYP3A4 (Figure 3E–H). We subjected CBN-11-OH to human liver microsomes to analyze secondary metabolism (Figure S1). We also used Bio Transformer 3.0 software^22^ which helped to predict possible biotransformed metabolites from CBN-11-OH and CBN-1′–OH (Figure S1). Most of the products involve hydroxylation (single or at multiple sites) around the fused benzene ring or at different positions in the aliphatic tail. The predicted products of identified *m*/*z* peaks eluting at 4.65 min, which correspond to the *m*/*z* of a single hydroxylation (343.19) with a single hydrogen adduct [M + H]+ and also an *m*/*z* (358.20), which corresponds to hydroxylation at two different sites [M + H – H]+ (Figure S3A,B).

**Figure 3:**
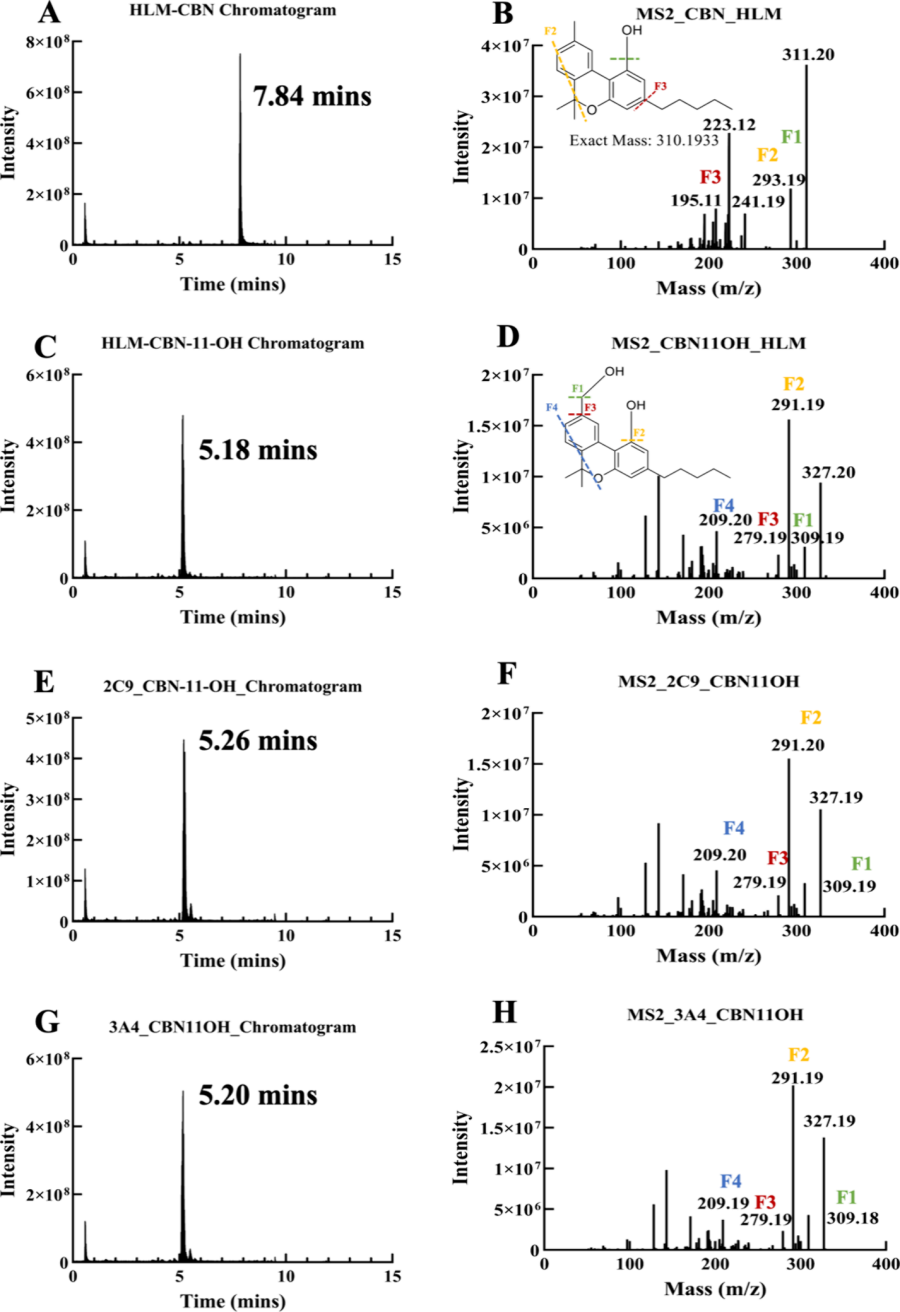
LC and MS2 fragmentation analysis of CBN and CBN-11-OH by human liver microsomes, CYP2C9 and CYP3A4. (A) CBN peak is detected at 7.78 min when subjected to metabolism by HLM, and the respective MS2 fragmentation of CBN is represented in (B). The CBN-11-OH peak is detected at 5.23 min in (C), and the respective MS2 fragmentation pattern of CBN-11-OH in (D). LC (E) and the MS2 patterns of CBN-11-OH formed from the metabolism of CBN by CYP2C9 are shown in (F). LC (G) and the MS2 patterns of CBN-11-OH formed from the metabolism of CBN by CYP3A4 are shown in (H).

CBN is partially metabolized in Phase 1 by CYPs and is further subjected to Phase II enzymes such as UDP glucuronosyltransferase (UGTs). Glucuronidation of these metabolites makes them highly hydrophilic (polar), subsequently leading to their elimination through urine.^23^ We report the glucuronidated products of CBN and CBN-11-OH (Figure S2). Our results indicate the formation of glucuronidated products of CBN with a peak at *m*/*z* 488.35 corresponding to mass with a hydrogen adduct [M + 2H]+ and a retention time of 5.09 min. We also identified the CBN-11-OH glucuronidation, with a retention time of 8.94 min and a peak at *m*/*z* 503.35 corresponding to mass with a hydrogen adduct [M + H] ^+^ (Figure S3C,D).

#### In Vivo Metabolism of CBN in Female Mice and by Human Liver Microsomes (HLM) Using Targeted Mass Spectrometry

After we identified the major metabolites of CBN upon direct metabolism by purified CYPs and HLMs, we developed a targeted mass spectrometry method for the quantification of these metabolites using synthesized and commercially purchased standards and measured the rate of formation of the metabolites. The details are provided in the Experimental Section below under quantitation of CBN metabolites by LC/MS-MS.

Using this targeted metabolomics method, we measured CBN and its metabolites generated during *in vivo* metabolism, where we fed CBN to female mice. We also measured the same metabolites during CBN metabolism by HLMs. We used two routes of CBN administration, intraperitoneal (*i.p*) and intravenous (*i.v*) and extracted plasma at two different time points, 0.5 and 2 h after treatment. Our results show that the amount of CBN available through the intravenous route of administration is 3.0 times higher than that available through intraperitoneal administration (Figure 4). The primary metabolite observed from CBN metabolism is 11-hydroxy CBN. CBN-1′–OH and Cannabinol *p*-quinone (CBN-PQ) are also formed in both routes of administration. CBN-11-OH formation is ∼ 89 times higher than CBN-1′–OH and ∼55 times higher than CBN-quinone after 0.5 h in the IV route of administration. In the i.p. route of administration, CBN-11-OH formation is ∼82-fold higher than CBN-1′–OH and ∼90-fold higher than CBN-quinone after the same time. Between 0.5 and 2 h after administration with CBN, there is a ∼ 3-fold reduction in the availability of CBN through IV and a 3-fold reduction in CBN-11-OH. CBN is reduced by 3-fold in the IP route, and CBN-11-OH is reduced by 2-fold. CBN-PQ is another metabolite that forms second to CBN-11-OH in IV-mediated administration, and there is a 9-fold reduction in the levels between 0.5 and 2 h. CBN-1′–OH is reduced by 3-fold in the same duration.

**Figure 4:**
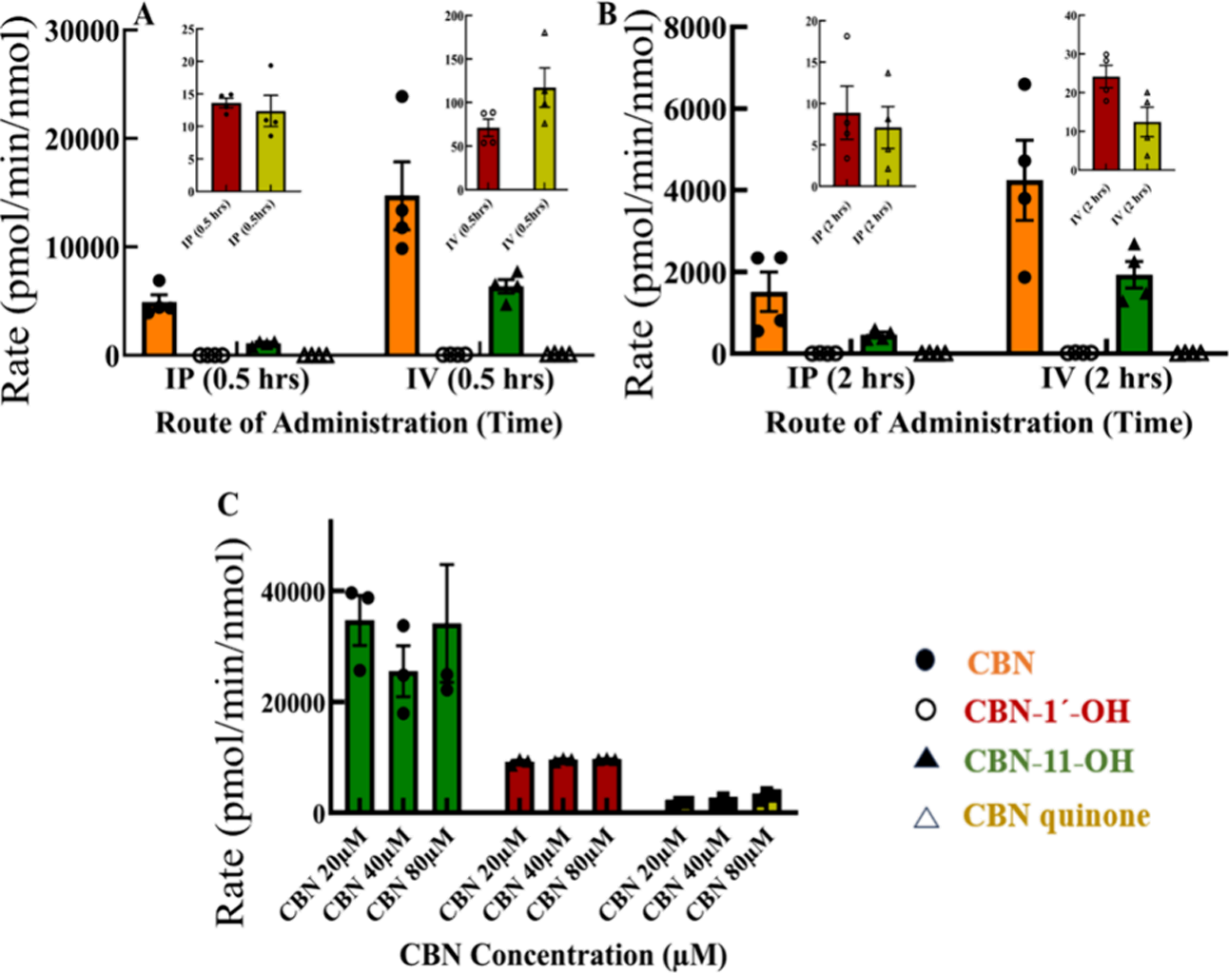
Quantitative targeted LC/MS analysis of CBN metabolism from mice plasma samples. The results show the CBN availability and the formation of CBN-11-OH as the primary metabolite from CBN-fed mice plasma samples after (A) 0.5 h and (B) 2 h of treatment through IP and IV routes of administration. The insets show the formation of the CBN-1′-OH and CBN-PQ (C) CBN is subjected to metabolism by human liver microsomes at three different concentrations and formation of CBN-11-OH (green), CBN-1′–OH (red) and CBN-Quinone (yellow).

Upon comparison of CBN metabolism in plasma samples extracted from CBN-fed mice with HLM-mediated metabolism of CBN at selected concentrations, we report a similar trend in the formation of metabolites. Both the data show that CBN-11-OH is the primary product being formed, followed by CBN-1′–OH. Another interesting observation was the extremely high metabolism rates for CBN by the HLM samples, with rates of metabolite formation reaching as high as 3.4 × 10^4^ pmol/min/nmol for CBN-11-OH. Compared to this, CBN-1′–OH and CBN-quinone rates were lower. CBN-1′–OH rates across different CBN concentrations were 9–9.4 × 10^3^ pmol/min/nmol, and quinone was further down by approximately three times the value of CBN-1′–OH. The human liver microsome-mediated CBN metabolism data at different concentrations of CBN also show a concentration-dependent increase in CBN-1′–OH and CBN-quinone. Although they were much lower in concentration than CBN-11-OH, this also corroborates our observation of the mice’s plasma metabolism.

#### Targeted LC/MS/MS and Quantitative Estimation of CBN Metabolites Formed By CYP2C9, CYP3A4, and CYP2D6-Mediated Metabolism

We employed targeted mass spectrometry to elucidate the mechanism and rate of formation of specific CBN metabolites. We measured the rate of formation of CBN-1′–OH, CBN-11-OH and CBN-quinone using three purified CYP450s, 2C9 and 2D6 in Nanodiscs and pooled HLM.^24^ In these experiments, we used purified Cytochrome P450 reductase (CPR) reconstituted with an 8:2 POPC–POPS lipid mixture and purified CYP2C9. The CBN metabolism by CYP2D6 was done in a nanodisc. The metabolism experiments were performed in the concentration range of 5–80 μM as these reflect physiological concentrations and are within the limit of CBN solubility.

The individual human cytochrome P450s were expressed and purified as discussed in the Experimental Section below. Our results show that CYP2C9-mediated metabolism of CBN led to the formation of CBN-1′–OH and CBN-quinone, and CBN-11-OH. We fit the total product rate which exhibited Michaelis–Menten kinetics. Interestingly, CBN-11-OH production, indicates atypical P450 kinetics (Figure 5B). Previous reports of phytocannabinoids show both Michaelis–Menten and atypical kinetics.^6,7^ Among the main metabolites, CBN-11-OH shows the highest rate of formation. CBN-1′–OH shows a Vmax of 12.1 pmol/min/nmol, which is lower than CBN-11-OH, showing a Vmax of 34.2 pmol/min/nmol. Also worth noting is that although the formation rate of CBN-11-OH is higher than CBN-1′–OH, the Km of CBN-1′–OH (2.3 μM) is much lower than CBN-11-OH (51.6 μM) (Figure 5E) The estimated value of Vmax for CBN-quinone is 4.4 pmol/min/nmol, and Km is 2.5 μM. Our targeted metabolomics using CYP2D6 and CBN also indicated that CBN-11-OH is the major product compared to CYP2C9. We also detected the formation of CBN-quinone, although it was also at a lower rate than CBN-11-OH. Our results with CYP2D6-mediated metabolism of CBN showed that CBN-11-OH and CBN-quinone show different kinetics of formation. CBN-11-OH shows a Vmax of 6.6 pmol/min/nmol, which is higher than CBN-quinone, which shows a Vmax of 5.6 pmol/min/nmol (Figure 5A–E). The respective Km value for CBN-11-OH is 14.84 μM, and for CBN-quinone, it is undertermined. Surprisingly, we did not detect the formation of CBN-1′–OH with CYP2D6. We do not report significant metabolism by CYP3A4 (Figure 5).

**Figure 5:**
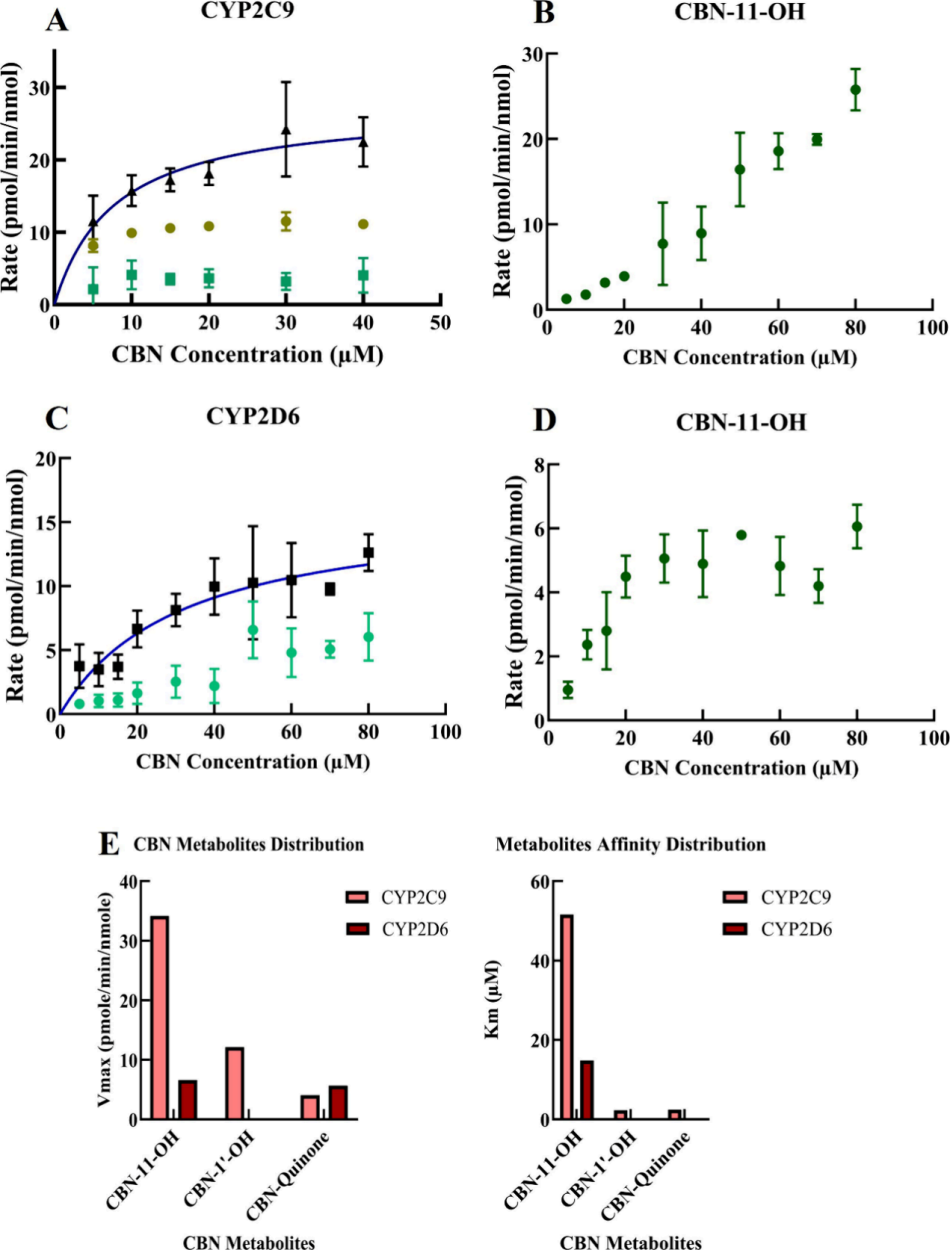
Quantitative LC/MS/MS measuring the kinetics of CBN metabolism by CYP2C9 and CYP2D6. The substrate concentration of CBN was 5–80 μM. The metabolism by CYP2C9 shows the formation of (A) CBN-1′-OH and CBN-quinone, and the total product rate (B) CBN-11-OH. Also, the kinetics of the formation of CBN-11-OH and the other metabolites are different. (C) CYP2D6-mediated metabolism also shows the formation of CBN-quinone, and the total product rate (D) CBN-11-OH. We detected no formation of CBN-1′-OH-in CYP2D6-mediated metabolism. (E) Comparative Vmax and Km values for CYP2C9 and CYP2D6 with respect to different metabolites as estimated. The Vmax and Km values were estimated using GraphPad prism. The error bars represent the SEM value of the respective samples, where *n* is *n* = 3.

### In Silico Studies on CBN Interaction with CYP2C9

To elucidate the putative binding mode of CBN within the CYP2C9 active site, which may facilitate the formation of the dominant metabolite 11-hydroxy CBN (Figure 6A), we performed docking of CBN to the CYP2C9 active site (PDB ID: 1R9O) using the cocrystallized ligand flurbiprofen as a reference. Additionally, we also docked THC to compare its binding modes and stability within the CYP2C9 active site. Our goal was to identify a binding pose for CBN and THC that would likely lead to their primary metabolites as produced by CYP2C9, CBN-11-OH and THC-11-OH, respectively. To choose the best pose, we applied two criteria. First, we measured the distance between the hydroxylation target carbon atom(s) in each cannabinoid (CBN-C11, THC-C11) and the Fe atom of the heme. The second metric involved the root-mean-square deviation (RMSD) between the center of mass (COM) of the ring systems of each cannabinoid and the COM of the crystal ligand, flurbiprofen, to ensure alignment within the active site. The results of these two metrics for each docked pose are shown in Figure S8. The docking energy for the respective compounds are in Table S1. We selected the best pose for each cannabinoid based on a short target carbon-to-Fe distance and strong alignment with the cocrystallized ligand (low RMSD). The best docked pose of CBN is presented in Figure 6B, demonstrating a favorable alignment with the cocrystallized ligand, flurbiprofen. Notably, the target carbon atom (C11) of CBN is positioned 4.55 Å from the heme group’s iron (Fe) atom. In the best docked pose of THC, THC-C11 is also positioned close to the heme group’s iron atom, 4.62 Å and THC is well aligned with the crystal ligand (Figure S8A,B). Subsequently, we simulated the best docked pose (pose 1) of CBN and THC for 1000 ns to further evaluate their stability within the CYP2C9 binding site (Figures 6C–G and S8C–F).

**Figure 6:**
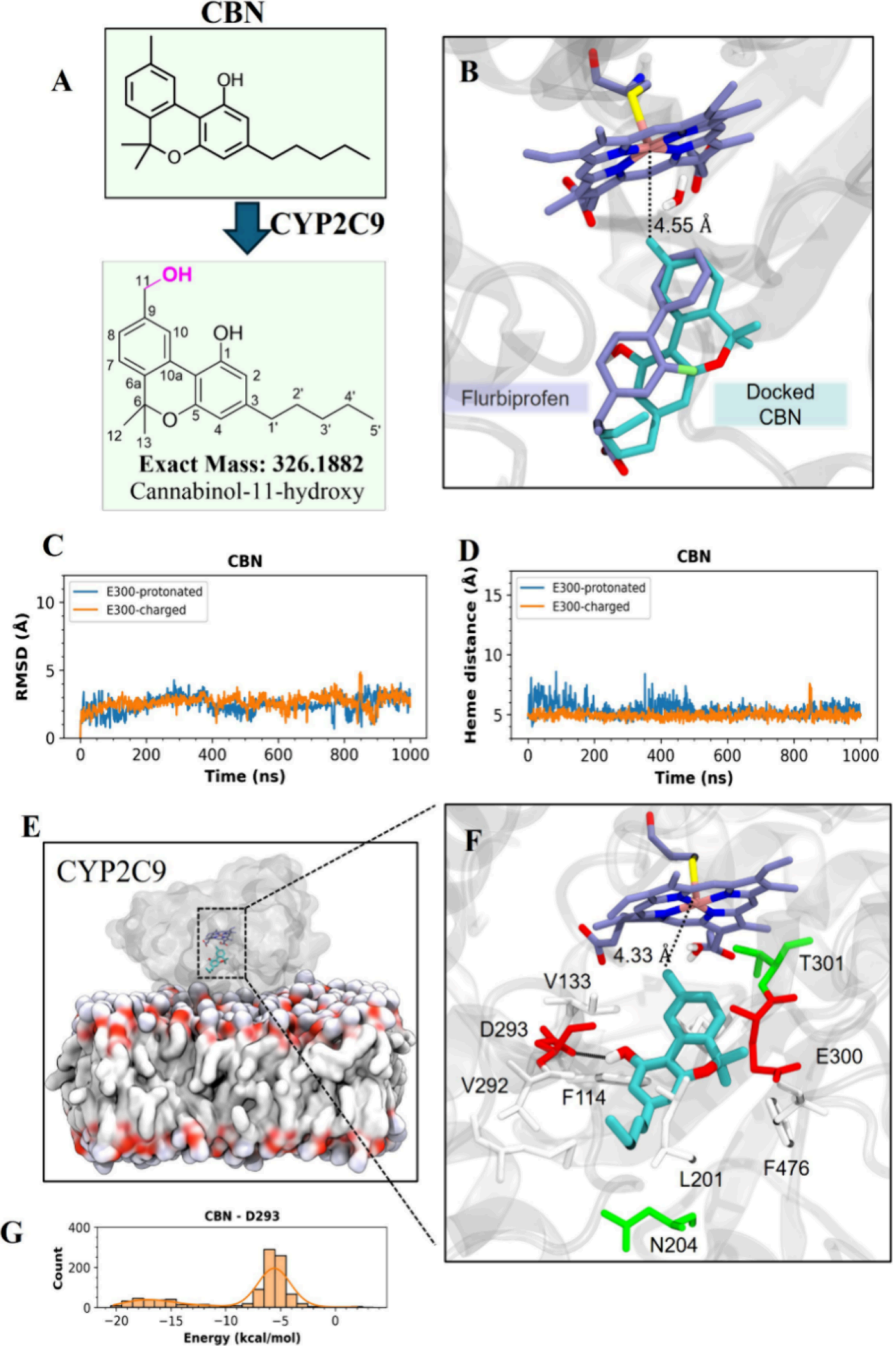
Putative binding mode of CBN to CYP2C9, which may lead to the production of CBN-11-OH. (A) Cannabinol metabolized by CYP2C9 to CBN-11-OH (B) Selected docked pose of CBN in the CYP2C9 active site. The crystal ligand, flurbiprofen, is depicted in lavender (carbon), while docked CBN is shown in cyan (carbon). The distance between C11 and the Fe atom (pink) is represented by a black dashed line. Fluorine, oxygen, sulfur, nitrogen, and hydrogen atoms are colored green, red, yellow, blue, and white, respectively. C418 and the water molecule coordinating the heme group are explicitly depicted. (C) RMSD of CBN throughout MD simulation. (D) CBN-C11’s distance to the Fe atom throughout MD simulation. (C, D) Results from two CYP2C9 models, one with protonated E300 and one with charged E300, are shown in blue and orange, respectively. (E) Representative snapshot of the MD setup with a docked CBN in the active site. CYP2C9 and the membrane are shown as surface representations. CBN and heme group are shown in cyan and lavender. (F) A representative snapshot of CBN from the MD simulation, highlighting some of the critical residues surrounding CBN. Hydrophobic residues are colored white. Water molecule coordinating the heme iron is shown in ball-and-stick representation. Hydrogen bonds are shown as black dashed lines. The distance between the C11 and the Fe atom (shown in pink) is also shown as a black dashed line. (G) Histogram of the total of nonbonded energies between CBN and D293 collected at every ns of MD simulation.

We simulated two cannabinoid-bound CYP2C9 systems: one with a protonated E300 and the other with a charged E300, based on the predicted p*K*_a_ values of E300, which were 7.64 in the AlphaFold-predicted CYP2C9 model and 5.77 in the crystal structure (PDB: 1R9O) (Figure 6E). CBN (Figure 6C) and THC (Figure S8C) were both found to be stably bound in both the E300-protonated and E300-charged CYP2C9 systems, as indicated by RMSD values. To assess the proximity of the hydroxylation target carbon atoms of cannabinoids to the heme group during the MD simulations, we measured the distances between these carbon atoms—CBN-C11, THC-C11, which were nearest to the heme group—and the iron (Fe) atom of the heme. In the E300-charged CYP2C9 systems, both CBN-C11 and THC-C11 remained approximately 5 Å from the heme group throughout the simulations (Figures 6D and S8D). We also analyzed the residues that interacted with cannabinoids for more than 20% of the time during MD simulations, accompanied by a representative snapshot illustrating the key residues surrounding the cannabinoids (Figures 6F and S8E,F). Throughout the simulations, CBN interacted with multiple hydrophobic residues in the CYP2C9 active site (Figures 6E,F and S8E,F). Notably, the hydroxy group of CBN formed a hydrogen bond with D293 for 18.5% of the simulation time (Table S2) (Figure 6F). The interaction energy distribution between CBN and D293 predominantly ranged around −6 kcal/mol while also sampling energies between −15 and −20 kcal/mol (Figure 6G). Notably, the D293 residue is highly conserved among CYPs, and its mutation to Ala (D293A) in CYP2C9 has been reported to decrease CYP2C9 activity by 90%.^25^ Significant attractive interactions form between CBN and D293, likely due to consistent and close hydrogen bond interactions, highlighting the importance of this key residue in CBN metabolism by CYP2C9 ^25^ Similarly, THC was also surrounded by hydrophobic residues (Figure S8F) and formed hydrogen bonds with D293 for 26% of the simulation time. Lastly, we estimated the binding free energies of cannabinoids to CYP2C9 by selecting a representative snapshot from the MD simulations and utilizing the Molecular Mechanics Generalized Born Surface Area (Prime MM-GBSA) tool in Maestro.^26^ The resulting binding free energies were as follows: CBN (−44.58 kcal/mol), THC (−53.71 kcal/mol). The difference in MM-GBSA estimated binding free energies between CBN and THC aligned with the experimentally measured *K*_i_ values,^27^ with IC50 values of 0.42 ± 0.13 and 0.19 ± 0.13 μM, respectively.

#### Evaluation of CBN and Its Metabolites for Anti-Inflammatory Activity in Microglial Cells

Microglial cells are macrophages in the central and peripheral nervous system that act as the first line of defense against pathogens.^28^ They respond to inflammation, cell and tissue damage, and injury to the brain, which includes the secretion of cytokines and chemokines.^29^ We have used the BV2 microglial cell line to assess the various pro (IL-6, NO and LDH) and anti-inflammatory (IL-10 and Arginase1) markers under lipopolysaccharide (LPS) stimulation in the presence and absence of CBN and its metabolites.^6^ The cell titer-blue (CTB) cell viability assay showed that cells are viable under the treatment with the molecules. LDH cytotoxicity assay showed that LDH production was reduced in cells treated with CBN and its metabolites in cells treated with LPS (Figure S7A,B). The concentration range used in the studies aligns with previous literature, which has shown that the cannabinoids are in low micromolar range levels in human plasma, 0.5 h after administration.^6,30,31^ We assessed the pro-inflammatory and anti-inflammatory markers in LPS-stimulated microglial cells. In cells treated with CBN and CBN-11-OH, there is a slightly lower production of NO (pro-inflammatory marker) at 5 μM (Figure 7A,B). For pro-inflammatory interleukin IL-6, at a concentration of 5 μM, CBN and CBN-11-OH show a reduction in IL-6 levels. CBN-1′–OH also showed a decrease in IL-6 production (Figure 7C,D). In cells treated with CBN and its metabolites, there is a concentration-dependent increase in IL-10 levels in CBN and CBN-11-OH-treated cells, which is reversed in the case of cells treated with CBN-1′–OH. Still, the level of IL-10 is not higher than that of LPS-stimulated cells without any molecules (Figure 7E,F). Arginase 1 is an enzyme expressed in immunosuppressive environments and is reported to reduce NO levels and subsequent pro-inflammatory interleukins like IL-6. We show that there is a slight increase in Arginase1 levels when cells were treated with CBN and CBN-1′–OH, although it showed mixed results when cells were treated with CBN-11-OH (Figure S7C).

**Figure 7:**
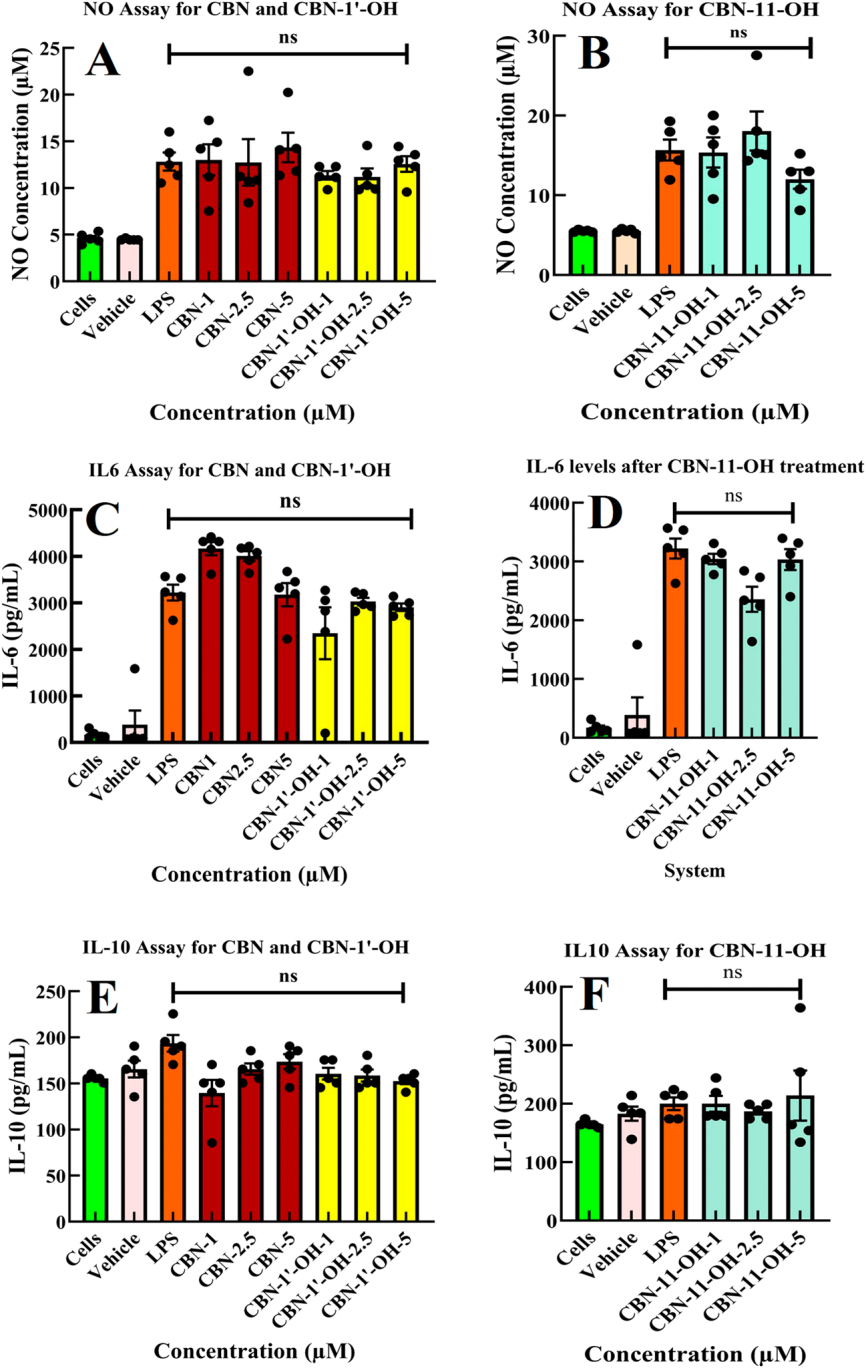
Effect of CBN and hydroxy metabolites of CBN on various inflammatory markers like NO, IL-6, and IL-10 on LPS-stimulated BV2 microglial cell lines. (A) and (B) NO levels (μM) as detected through NO assay from LPS-stimulated BV2 cells. There is no detectable significant (ns) change in the NO levels in CBN, CBN-11-OH, and CBN-1′-OH treated (at 1, 2.5, and 5 μM concentrations) BV2 cells compared to cells with only LPS treatment. (C) and (D) Changes in IL-6 levels and (E) and (F) changes in the IL-10 levels. All the markers have been examined from the same batch of supernatant. For statistical analyses, we used one-way ANOVA with Brown–Forsythe and Welch ANOVA tests. For multiple comparisons, we used the Dunnett T3 test and reported p-values with less than 0.05 as significant and ns implies not significant. The number of samples for each condition was *n* = 5. We used the GraphPad Prism 10 software to estimate the same.

#### Comprehensive Analysis of CB1 and CB2 Receptor Modulation by CBN and Its Metabolites

To investigate the modulation of cannabinoid receptors CB1 and CB2 in response to CBN and its metabolites CBN-1′–OH, CBN-11-OH, and CBN-quinone, we used a cell-based G protein nano BRET assay.^32^ We first employed this assay in agonist mode. Transfected cells were stimulated with 10 μM of each compound, and ΔBRET ratios were calculated as an index of receptor activation in the presence of representative Gα proteins to obtain a fingerprint of G protein activation for each receptor (Figure 8A,B). These experiments showed a partial agonist effect of CBN-1′–OH at the CB1 receptor when activating Gαo. Later, we obtained concentration–response curves for Gαo activation in response to the application of CBN and each of its metabolites for both CB1 and CB2 receptors (Figure 8C). The full agonist CP-55940 was used as a positive control. This data confirmed partial agonism of CBN-1′–OH on the CB1 receptor, detectable only at high concentrations of the ligand. The partial agonism behavior of Δ9-THC has been previously shown in our work using docking and simulation.^33^

**Figure 8:**
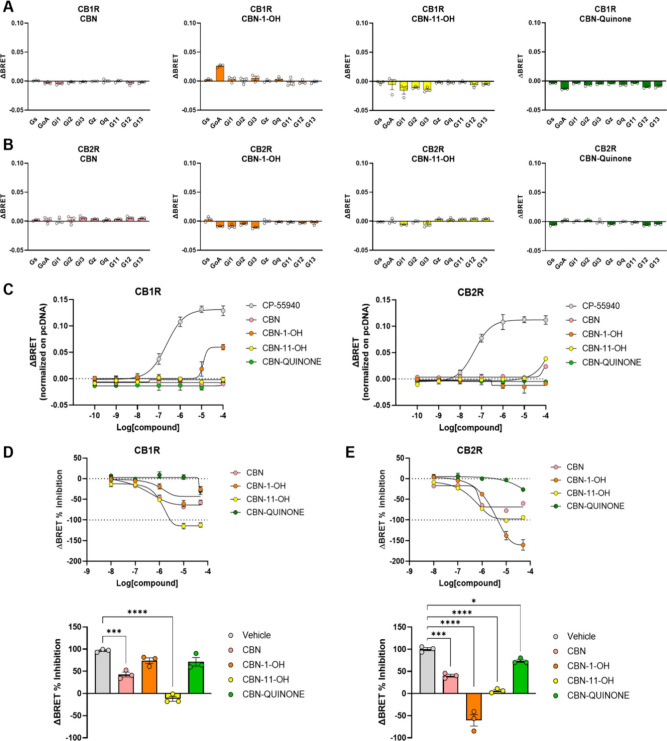
Analysis of CB1R and CB2R activity after treatment with CBN and its metabolites. G protein nanoBRET assays were used to obtain a fingerprint of G protein activation by CB1R (A) or CB2 (B) receptors in response to treatments with 10 μM of indicated compounds. *N* = 3. (C) Concentration–response curves of GoA activation by CB1R and CB2R after application of indicated CBN metabolites. The full agonist CP-55940 was used as a reference (pEC50 = 6.64). *N* = 3. (D) Concentration–response curves of CB1R inhibition after pretreatment with 1 μM CP-55940 and application of indicated CBN metabolites (top panel). The ΔBRET ratio was calculated for an additional 100 s. In the lower panel, we quantified the signal inhibition compared to the vehicle at 50 μM of CBNs applied. One-way ANOVA with Dunnett’s comparison to vehicle. *N* = 3, ***p < 0.001; ****p < 0.0001. (E) Concentration–response curves and quantification of inhibition at 50 μM CBN and its metabolites applied for CB2 receptor. One-way ANOVA with Dunnett’s comparison to vehicle. *N* = 3 *p < 0.05; ***p < 0.001; ****p < 0.0001.

Next, we tested the ability of each compound to act as antagonists at CB1 and CB2 receptors. For this, we pretreated transfected cells with 1 μM of orthosteric full agonist CP-55940, we recorded the signal for 100s, then we applied increasing concentrations of each CBN metabolite and calculated the BRET ratio for an additional 100s. The concentration–response curves obtained for the CB1 receptor showed a competitive inhibition of signal, indicating that some of the CBN metabolites bind at the orthosteric site. We found a significant but partial inhibition of CB1 activation when we applied 50 μM CBN and a complete ablation of the BRET signal with 50 μM CBN-11-OH (Figure 8D). Similarly, we obtained concentration–response curves showing significant inhibition of the CB2 receptor by application of all the CBN compounds at 50 μM (Figure 8E). The curves indicate partial inhibition by CBN and CBN-quinone, complete inhibition by CBN-11-OH, and an inverse agonist effect by CBN-1′–OH at CB2, reducing the signal below the basal constitutive activity of the receptor (Figure 8E).

#### CBN and CBN-11-OH Induced Activation of Sensory Neurons

CBN is found in extracts of the plant and is the stable degradation product of Δ^9^-THC following air oxidation.^34^ Therefore, it is expected that CBN will be present in increased amounts following the aging or heating of and/or Δ^9^-THC. Despite its apparent abundance, there is limited knowledge about the structure, abundance and physiologic consequences of its metabolite(s) in the nervous system.^4,5^ Importantly, a major metabolite of CBN is CBN-11-OH, reported to have a differential binding affinity to the cannabinoid receptor CB_1_R than the parent compound CBN.^10^ Little is known about the physiology of CBN-11-OH or its potential action on sensory neurons.^36,37^

While CBN has been studied for its ability to activate/inactivate sensory neurons in the pain pathway^30,31^ and members of the TRP family of channels in heterologous systems (11, 32)­Physiological studies of its metabolite, CBN-11-OH, are largely absent. Given our findings that CBN-11-OH is the primary metabolite of CBN, we performed the physiologic characterization of CBN-11-OH-induced activation. [Ca^2+^]_i_ responses of 38.9 ± 4.3% (*n* = 56), which did not differ from the peak values directed by CBN-11-OH, 31.6 ± 2.8% (*n* = 29; ns, not significant) (Figure 9C). In contrast (Figure 9D), CBN-11-OH activated a surprisingly lower percentage of DRG neurons (5.4 ± 1.1%) as compared with CBN (29.3 ± 4.9%) (*p* < 0.0001). Moreover, we found that CBN-11-OH induced calcium responses more rapidly than those observed with CBN (Figure 9E), with CBN-11-OH directing less time to peak response (22.8 ± 2.2 s) compared with CBN (40.7 ± 4.8 s).

**Figure 9:**
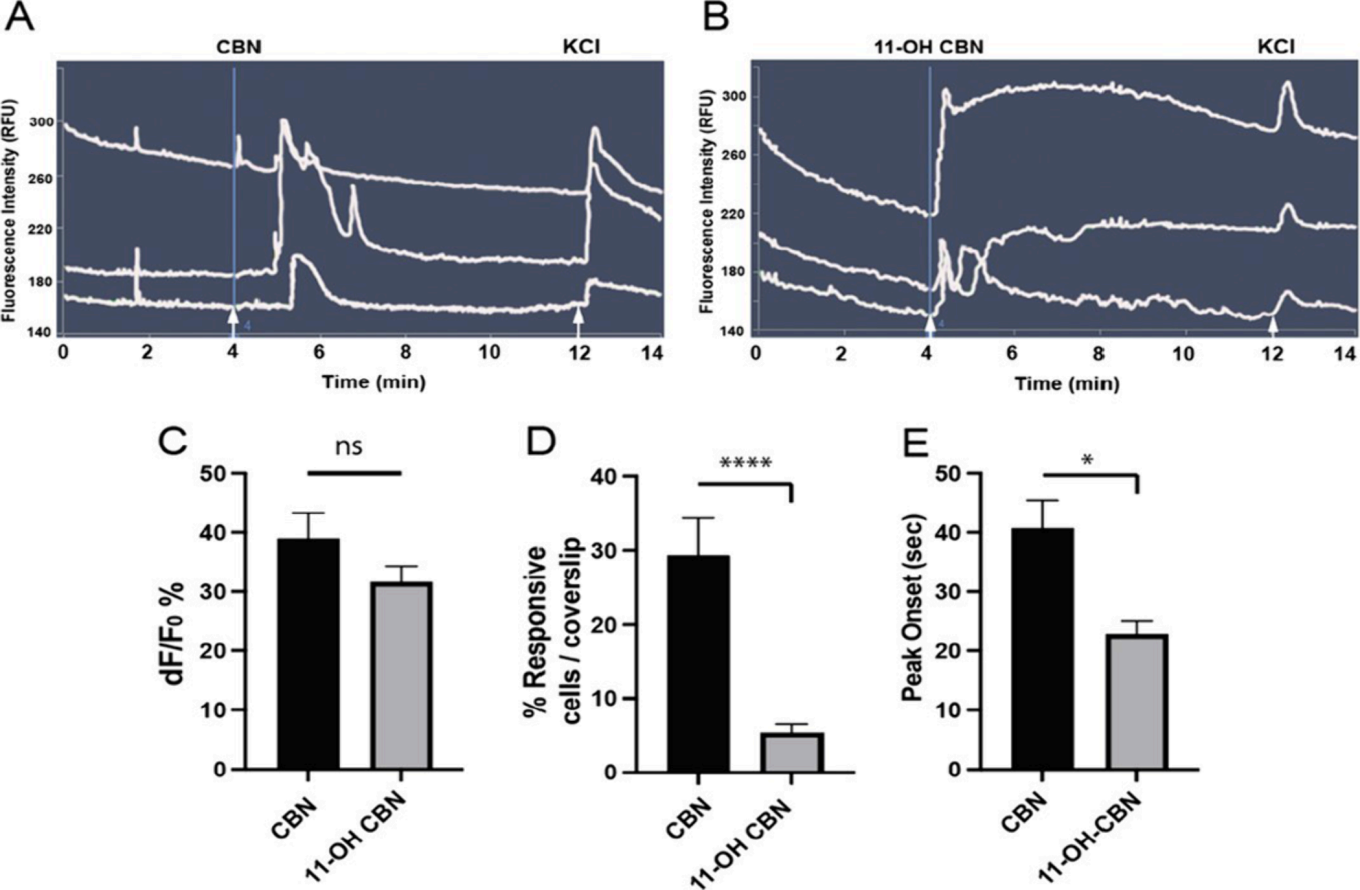
CBN and 11-OH CBN activate DRG neurons with similar response magnitudes but differ in their percentage of neurons activated and time to peak response. Calcium response traces of representative cultured mouse DRG neurons following application of CBN (A) and 11-OH CBN (B) 50 μM × 15 s (arrow). While CBN and 11-OH CBN response magnitudes did not differ (ns = not significant) (C), 11-OH CBN activated a lower percentage of DRG neurons (****p < 0.0001) (D) and reached peak responses more quickly (*p = 0.011) (E) as compared to CBN. Mean value ± SEM (CBN: *n* = 59; 11-OH CBN: *n* = 29, two-tailed *t* test, Prism GraphPad).

## Discussion and Conclusions

The pharmacological potential of phytocannabinoids continues to be an expanding area of scientific research given their physiological significance. Recently, attention has shifted toward minor cannabinoids, which are nonpsychoactive and exhibit a range of therapeutic properties. CBN is one such minor cannabinoid that occurs naturally and is also formed through the oxidation of THC, the primary psychoactive component of cannabis. CBN is nonpsychotropic and has been traditionally used as a sleep aid.^38^ While early studies suggested that CBN possesses analgesic properties comparable to aspirin in inflammatory pain models,^39^ there remains a limited understanding of its broader antinociceptive effects and its potential to mitigate hypersensitivity.^2,40^ More recently, CBN has been identified as an inhibitor of sodium channels (Nav1.7) in DRG neurons, highlighting its potential role in the treatment of neuropathic pain.^35^

It is well established that cannabinoids such as THC, CBN, CBG, and CBC are metabolized by cytochrome P450 enzymes.^6,7,17^ As primary phase 1 drug-metabolizing enzymes, it is less widely recognized that their primary metabolites may be bioactive and contribute to the pharmacological effects of the parent cannabinoid. Previously we have shown that CBG and CBC formed bioactive downstream metabolites that exhibited anti-inflammatory properties.^6,7^ In this comprehensive study, we demonstrate CBN metabolism by cytochrome P450 enzymes and identified the binding modes of CBN in the active site of CYP2C9 using MD simulations. These CBN metabolites exhibit bioactivity and interact with receptors and neurons, highlighting their potential pharmacological significance as antinociceptive/analgesic- compounds.

We report the direct metabolism of CBN by cytochrome P450 enzymes, identifying its metabolites using LC-MS/MS. The primary metabolite, CBN-1′–OHCBN-11-OH, along with CBN-1′–OH and cannabinol quinone, were detected. Plasma extractions from CBN-treated mice were concordant with the metabolites formed during human liver microsome-mediated metabolism. Fragmentation patterns from LC-MS/MS were validated against synthesized standards. We report that CBN-11-OH is formed at higher rates compared to CBN-1′–OH and CBN-*p-*quinone both in vitro and in vivo systems, which further corroborates that CBN-11-OH is the major metabolite formed from CBN metabolism. The overall rate of metabolism of CBN is slower than previously reported rates for CBG and CBC by human cytochrome P450 and can be attributed to the chemical stability of CBN due to its aromatic rings.^16^ While previous studies identified CBN-11-OH and CBN-8-OH via GC-MS, we report the novel formation of CBN-1′–OH and cannabinol quinone, demonstrating that cannabinoid quinones can be endogenously produced during CBN metabolism^17^ Quinones have diverse biological effects, including acute cytotoxicity, immunotoxicity, and carcinogenesis, but they can also promote cytoprotection by stimulating detoxification enzymes, reducing inflammation, and altering redox balance. Additionally, a quinone derived from CBD has been explored as a potential cancer therapy.^41^

We report a higher amount of CBN availability in mice plasma after 0.5 and 2 h, respectively, through the intravenous route of administration compared to the intraperitoneal route. This contradicts our previous observation, where CBG and CBC underwent faster metabolization through the intraperitoneal route of administration.^7^ Drugs administered intraperitoneally have a similar pharmacokinetics to oral administration of drugs as the drugs enter through the mesenteric vessels, which lead them directly into the portal vein, and hence, they pass through the liver. As a result, this route leads to the hepatic metabolism of the drugs before reaching the systemic circulation.^42^ We observed a reduction in levels of both CBN and its metabolites to varying degrees over a period of 2 h in the mice plasma, implying CBN metabolites are also metabolized further or converted to other metabolites. Previous studies on other cannabinoids like cannabidiol, tetrahydrocannabinol and cannabichromene have all reported similar observations.^7,43^

Most of the phytocannabinoids produce oxidized products like hydroxy and epoxides by human cytochrome P450s, which undergo glucuronidation by UGT enzymes before being excreted.^14^ We showed that further metabolism of CBN and CBN-11-OH by human liver microsomes led to the detection of glucuronidated products. Previous studies have shown that only specific UGTs show sufficient activity toward phytocannabinoids like THC, CBN and CBD.^14,44,45^ A similar study analyzed the glucuronidation of CBC, CBG, and CBN using a suspension of human hepatocytes and showed that these molecules do form glucuronidated products along with hydroxylation.^44^

We further probed which human CYPs specifically metabolized CBN using recombinantly purified human CYPs stabilized in nanodiscs. Our results using targeted and untargeted metabolomics showed that CYP3A4 (major drug metabolizing enzymes) did not metabolize CBN while CYP2C9 and CYP2D6 metabolized CBN. Metabolism by CYP2C9, led to the formation ofCBN-11-OH, CBN-1′–OH and CBN *p-*quinone with varied rates. CYP2C9 active site analysis shows that it is capable of binding multiple ligands.^46^ This shows that the same substrate can form multiple metabolites at different rates, showing varied kinetic patterns. This makes it imperative to study drug metabolism with a broad range of CYPs, helping not only in drug design but also in understanding drug metabolism at a larger scale. With regards to CYP2D6 mediated metabolism of CBN, we only detected both CBN-11-OH and CBN-quinone and not CBN-1′–OH.^47^

Multiple studies have demonstrated that CYP2C9 plays a key role in metabolizing cannabinoids, particularly THC.^17^ Therefore, we aimed to investigate the binding dynamics of CBN and THC within the active site of CYP2C9 and to elucidate the underlying rationale for the formation of CBN-11-OH and THC-11-OH as the predominant metabolites (Figure 6). The findings highlight the significant roles of hydrophobic residues and the critical contribution of residue D293 within the CYP2C9 active site.

First, we used molecular docking to identify the best binding pose of CBN and THC to CYP2C9 active site (Figures 6B and S8A–C). To select the best pose from the docked complexes for further assessment with MD simulations, we used the alignment of CBN and THC with the native ligand (ibuprofen) and then measured the distances between the C11 of CBN and THC and Fe atom of the heme (Figure S8A–C). In both CBN (4.55Å, Figure 6B) and THC (4.62Å, Figure S8B), C11 was within 5Å. This explains the potential hydroxylation of this carbon atom and why, subsequently, C11-OH forms readily for both CBN and THC as the primary product. Crystal structure of CYP2C9 indicated that the active site is prone to binding acidic lipophilic substrates.^46^ Generally, phytocannabinoids are lipophilic in nature. The CYP2C9 active site has a rich core of hydrophobic residues. F114, which points toward the active site, is well-positioned for interactions with substrates. Other than these residues, L102, L208, L362, L366 and F476 all play a major role in substrate binding within the active site of 2C9.^46^ When we analyzed our simulation results measuring the probability of interaction with various residues within the active site, we noticed that L362, L366 and F476 all had decent interaction probability with CBN, although we identified E300, V113 and D293 as well to stand out as having stronger interaction probabilities with CBN (Figure S8E). D293 is close and is in between F110 and F114 and is well ordered. It forms hydrogen bonds with the nitrogen of I112.^46^ Since F114 is already an important residue in the active site channel, residues surrounding F114 might also play an important role in substrate binding. E300, which is another acidic residue present within the active site of the enzyme and points toward the active site, has relatively higher flexibility when no ligand is bound.^46^ Our interaction pattern with CBN showed E300 to have a major interaction with CBN during simulation (Figure S8E). When we analyzed the binding pattern for THC, D293 and E300 also showed plausible interaction probability, with D293 showing a higher probability compared to what it showed with CBN (Figure S8E,F). F100 also showed interaction with THC contrary to CBN (Figure S8E,F). The remainder of the active site residues showed decent interaction probability. Upon further analysis, it showed that CBN and THC showed 18.5 and 26% hydrogen bond interactions with D293 during the simulations. Taken together, we modeled the putative binding pose of CBN to cytochrome CYP2C9 to explore the pathway leading to its major metabolite, CBN-11-OH. Cannabinoid metabolites are proposed to be bioactive because they retain structural and functional pharmacophores similar to their parent compounds, enabling interactions with biological systems. Many of these cannabinoid metabolites continue to target key receptors in the endocannabinoid system, including CB1 and CB2 receptors, thereby influencing physiological processes such as inflammation, pain modulation, and neuroprotection. Some metabolites possess distinct pharmacological properties, functioning as partial agonists, antagonists, or allosteric modulators of these receptors. For example, hydroxylated metabolites of CBD have been shown to exhibit anti-inflammatory, antioxidant, and neuroprotective effects.^48^ Other metabolites affect noncannabinoid targets, such as transient receptor potential (TRP) channels, peroxisome proliferator-activated receptors (PPARs), and enzymes involved in oxidative stress responses. In this study, we explored the bioactivity of CBN and its hydroxy metabolites, focusing on their anti-inflammatory properties, interactions with cannabinoid receptors, and effects on dorsal root ganglion (DRG) neurons.

To assess this, we measured pro- and anti-inflammatory markers in LPS-stimulated BV2 microglial cells treated with these molecules. Activated macrophages secrete inflammatory markers such as IL-6, IL-1, and NO, which are produced via the iNOS pathway—a key player in cytokine release during inflammation.^49^ Our findings indicate that CBN and its metabolites do not reduce these inflammatory markers, unlike CBG and CBC metabolites.^6,7^ Additionally, we examined Arginase 1, an enzyme that competes with nitric oxide synthase (NOS) for the substrate l-arginine. Our results showed no significant increase in Arginase 1 levels (Figure S7C) in LPS-stimulated BV2 cells, aligning with our observation of unchanged NO levels. Notably, a previous study reported that CBN exhibited anti-inflammatory effects at higher concentrations (15 μM), reducing markers such as TNFα, IL-1β, IL-6, and COX2. However, that study employed a very high LPS dose (500 ng/mL), whereas our experiments used a more moderate dose for LPS stimulation of 25 ng/mL.^50^

CBN interacts with both CB1 and CB2 cannabinoid receptors, though with differing affinities. It has a lower binding affinity for CB1 compared to Δ9-THC, resulting in reduced potency in CB1-mediated effects such as adenylyl cyclase inhibition.^4,51^ This weaker interaction likely explains its limited psychoactive properties, making it an attractive option as a sleep aid and pain reliever. In contrast, CBN exhibits comparable potency to Δ9-THC in CB2 receptor-mediated inhibition of adenylyl cyclase and is considered more active at CB2, which is primarily involved in immune modulation and anti-inflammatory effects.^4,51^ Overall, CBN acts as a partial agonist at both CB1 and CB2 receptors, with a stronger effect at CB2.^52^ Conversely, CBN-11-OH (interacts with cannabinoid receptors with greater potency, particularly at CB1. Studies have shown that CBN-1′–OH and CBN-11-OH act as partial agonists at CB1, with slightly lower potency than Δ9-THC, which may explain CBN’s central effects, including its neuropharmacological and hypnotic properties.^38^ At higher concentrations, CBN-11-OH also exhibits partial agonist activity at CB2.^38^ While prior studies assessed CBN and CBN-11-OH’s ability to activate cannabinoid receptors indirectly, radioligand binding assays have shown that CBN-11-OH can antagonize the effects of cannabinoid agonists on adenylyl cyclase inhibition in CB2-transfected CHO cells. In our study, we employed a BRET-based method to measure receptor activity. Our experiments demonstrated that CBN-1′–OH acts as a partial agonist for CB1 at high concentrations, specifically activating the Gαo protein. Antagonist mode studies further revealed that CBN and CBN-11-OH partially or fully suppress BRET signals, indicating complete receptor inhibition. Additionally, CBN and its metabolites function as partial or strong antagonists at the CB2 receptor.

CBN is comparable to aspirin in reducing pain in the inflamed hind paw of the rat^39^ and is an inhibitor of sodium channels (Nav1.7) in DRG neurons, with potential use as a pain drug.^35^ Further receptor studies have shown that CBN-11-OH has a higher affinity for CB1 (188.9 ± 38.3 nM) and CB2 (26.6 ± 5.5 nM) receptors. This metabolite is twice as potent as CBN against CB1 receptors and higher against CB2 receptors.^10^ We demonstrate here that the CBN metabolite, CBN-11-OH, is an important component of CBN-mediated analgesia in the body.

In summary, in this work, we highlight the metabolism of CBN into bioactive metabolites via cytochrome P450s primarily CYP2C9 and CYP2D6, leading to bioactive metabolites such as CBN-1′–OH, CBN-11-OH, and cannabinol quinone. CBN undergoes slower metabolism compared to other cannabinoids like CBG and CBC, possibly due to its aromatic structure. Its major metabolite, CBN-11-OH, forms at higher rates than other metabolites, likely due to favorable binding within the CYP2C9 active site. CBN is more stable in plasma, showing higher availability at 0.5 and 2 h postintravenous administration in mice. The presence of its glucuronidated metabolites suggests further biotransformation by UGT enzymes. Unlike CBG and CBC, CBN and its metabolites do not significantly reduce inflammatory markers in microglial cells at moderate doses. CBN has lower CB1 affinity compared to THC but exhibits similar potency at CB2, making it a promising candidate for immune modulation. CBN-11-OH is more potent at both CB1 and CB2 receptors, contributing to its stronger pharmacological effects than CBN. CBN and CBN-11-OH may contribute to the analgesic impacts, with CBN-11-OH being more potent in reducing pain and exhibiting prolonged effects as shown by the results in DRG neurons. Overall, the study highlights the importance of CBN metabolism in shaping its pharmacological profile, with implications for pain management and drug development.

## Experimental Section

### Materials

We purchased the antibiotics, ampicillin, chloramphenicol, and other reagents like isopropyl β-D-1 thiogalactopyranoside, Ni-NTA resin, and l-arabinose from Gold Biotechnology (St. Louis, MO, USA). The molecular biology materials like restriction enzymes and other reagents were purchased from New England Biolabs. We bought all other materials from Sigma and Fisher Scientific, like Lipopolysaccharides (-O17:B8). The drug-based substrates for the CYPs like Bromocriptine (mesylate) (Item No. 14598) and Ibuprofen (Item No. 15687-27-1), CBN-11-OH, (Item No. 30432-08-07) and Cannabinolic Acid (CBNA) (Item No. 2808-39-1) and the kits for NO (Item No. 780001), LDH (Item No. 601170) and MTT (Item No. 10009365) assays were purchased from Cayman Chemicals—the Mouse IL-6 uncoated ELISA kit from Thermo Fisher Scientific (Item No. 88-7064-88), IL-10 kit and Arginase1 kit from abcam (Item No. 269541). We purchased the human liver microsomes (HLM) vials of 20 mg/mL concentration available in 0.5 mL vials, mixed gender, pool of 50 suspended in 250 mM Sucrose. The specific content of CYP450 content was 0.369 nmol/mg of protein, cytochrome b5 content was 0.359 nmol/mg, and the rate of NADPH cytochrome c-reductase was 178 ± 8 nmol/mg protein/min.

The stock solutions of all the synthesized and purchased cannabinol-based compounds were dissolved in ethanol and stored at −80 °C until further use. We further prepared fresh diluted stocks of varied concentrations right before use. We maintained BV2 microglial cell lines at 37 °C and 5% CO_2_ incubators in Dulbecco’s Minimum Essential Media (DMEM) supplemented with high glucose (Cat No. 10–013-CV Corning Life Sciences). We further prepared the complete media for cell culture by adding 5% heat-inactivated Fetal Bovine Serum (FBS) and antibiotics Streptomycin (100 μg/mL) and penicillin (100 units/ml).

### Synthesis of CBN-Based Compounds

#### General Procedures

Unless otherwise noted, all chemicals were purchased from commercial suppliers and used as received. Acetonitrile (MeCN, HPLC grade), dichloromethane (CH_2_Cl_2_, HPLC grade), dichloroethane (DCE), benzene (ACS grade), and methanol (ACS grade) were used as solvents without further purification. 4-dimethylaminopyridine (DMAP), triethylamine (Et_3_N), acetic anhydride (Ac_2_O), selenium dioxide (SeO_2_), azobis­(isobutyronitrile) (AIBN), and silver acetate (AgOAc) were purchased from commercial sources and used without further purification. Cannabinol (CBN) was purchased from commercial sources and further purified by flash column chromatography (15:1 hexane: EtOAc). N-bromosuccinimide was purchased from commercial sources and further purified by recrystallization in water. Reaction temperatures correspond to the external temperature of the reaction vessel unless otherwise noted. Analytical thin-layer chromatography (TLC) was performed on Merck silica gel 60 F254 aluminum sheets. Visualization was accomplished with UV light and/or potassium permanganate (KMnO_4_). Retention factor (*R*_f_) values reported were measured using a 10 × 2 cm TLC plate in a developing chamber containing the solvent system described. Silicycle SiliaFlash P60 (SiO2, 40–63 μm particle size, 230–400 mesh) was used for flash column chromatography. ^1^H NMR spectra were obtained at 500 MHz and ^13^C NMR were obtained at 126 MHz. NMR spectra were recorded using a Bruker Avance III 500 MHz spectrometer equipped with BB CryoProbe or Varian/Agilent VNMRS 750 MHz Narrow Bore and were referenced to residual chloroform (7.26 ppm, ^1^H) and solvent chloroform-*d* (77.16, ^13^C). Chemical shifts are reported in parts per million (ppm), and multiplicities are indicated as s (singlet), d (doublet), t (triplet), q (quartet), p (pentet), m (multiplet), and br (broad). Coupling constants, *J*, are reported in Hertz. The University of Illinois Mass Spectrometry Laboratory performed mass spectrometry (MS). Electron Impact (EI+) spectra were performed at 70 eV using methane as the carrier gas, with a time-of-flight (TOF) mass analyzer. Electrospray ionization (ES+) spectra were performed using a time-of-flight (TOF) mass analyzer. Data are reported in the form of *m*/*z*. Infrared (IR) spectra were measured neat on a PerkinElmer Spectrum Two FT-IR ATR spectrometer. Peaks are reported in cm^–1^ with indicated relative intensities: s (strong, 0–33% T); m (medium, 34–66% T); w (weak, 67–100% T); and br (broad). Both CBN-1′–OH and CBN *p*-quinone, which were synthesized, were validated for their purity using HPLC analysis. Both of these compounds are reported to be more than 95% pure, as shown in our HPLC traces in the Supporting Information (SI). CBN and CBN-11-OH of purity greater than 98% were purchased from Cayman for other experiments.

### CBNQ (6,6,9-Trimethyl-3-pentyl-1H-benzo­[c]­chromene-1,4­(6H)-dione)

Compound number 1 in Figure 1 was synthesized as described here. Five mL of TBHP in PhMe (5.80 mmol, 3.6 eq, 3.8M) was added to a solution of SeO_2_ (0.322 mmol, 0.2 equiv) in 5.8 mL of DCE (0.28M) at 0 °C. After stirring for 2 min at 0 °C, a solution of CBN (500 mg, 1.61 mmol, 1 equiv) in 5.8 mL of DCE (0.28 M) was added dropwise slowly to the stirring solution. The solution was heated at 60 °C for 14 h. The crude reaction mixture was filtered through a plug of Celite and reconcentrated under reduced pressure. The natural material was purified by flash column chromatography using a gradient of 30:1 to 20:1 hexane: EtOAc). The pure product was isolated as a dark reddish oily residue (300 mg, 0.925 mmol, 57% yield).

*R*_f_– 0.60 (7:1 Hexane: EtOAc).

^1^H NMR (500 MHz, CDCl_3_) δ 8.18 (s, 1H), 7.17 (d, *J* = 7.9 Hz, 1H), 7.07 (d, *J* = 7.9 Hz, 1H), 6.48 (s, 1H), 2.43 (t, *J* = 7.8, 2H), 2.38 (s, 3H), 1.70 (s, 6H), 1.50–1.57 (m, 2H), 1.31–1.37 (m, 4H), 0.9 (t, *J* = 6.9, 3H).

^13^CNMR - ^13^C NMR (126 MHz, CDCl_3_) δ 186.72, 182.25, 151.40, 146.86, 137.97, 134.88, 133.61, 130.62, 127.70, 124.32, 122.75, 116.21, 81.42, 31.56, 28.57, 27.77, 27.44, 22.53, 21.50, 14.08.

HRMS– (ES+, *m*/*z*) [M + H]+ calcd. for C_21_H_25_O_3_ 325.1798; found, 325.1804.

IR– (ATR, neat, cm1): 2956 (m), 2924 (m), 2853 (m), 1737 (s), 1671 (s), 1651 (s), 1463 (s).

### 1′-Hydroxy-CBN (3-(1-hydroxypentyl)-6,6,9-trimethyl-6H-benzo­[c]­chromen-1-ol)

Compound number 5 in Figure 1 was synthesized as described here. To a solution of 1,1′-diacetoxy-CBN (170 mg, 0.414 mmol, 1 equiv) in 4.1 mL of MeOH (0.1M) was added K2CO3 (286 mg, 2.07 mmol, 5 equiv) in a single portion. The solution immediately changed from clear to a lime green solution. The reaction was monitored to completion by TLC and was found to be complete after 2 h. The organic phase was then washed with water and extracted with 4 mL of EtOAc (3x). The crude organic mixture was then dried over MgSO4, filtered, and purified by flash column chromatography using a gradient of 4:1 to 2:1 hexane: EtOAc. The product was isolated as a white solid (106 mg, 0.288 mmol, 70% yield).

*R*_f_– 0.10 (4:1 Hexane: EtOAc).

^1^H NMR (500 MHz, CDCl_3_) δ 8.22 (s, 1H), 7.15 (d, *J* = 7.9 Hz, 1H), 7.09 (d, *J* = 7.9, 1H), 6.58 (d, *J* = 1.8 Hz, 1H), 6.52 (d, *J* = 1.7 Hz, 1H), 5.93 (s, 1H), 4.58 (t, *J* = 6.3 Hz, 1H), 2.39 (s, 3H), 2.08 (s, 1H), 1.85–1.66 (m, 2H), 1.61 (s, 3H), 1.58 (s, 3H), 1.44–1.23 (m, 4H), 0.88 (t, *J* = 7.1 Hz, 3H).

^13^CNMR - ^13^C NMR (500 MHz, CDCl_3_) δ 154.86, 153.86, 146.08, 137.09, 137.07, 128.08, 127.41, 126.86, 122.74, 110.45, 108.49, 107.16, 77.63, 74.62, 38.44, 28.03, 27.27, 22.72, 21.68, 14.15.

HRMS– (ES+, *m*/*z*) [M + H]+ calcd. for C_21_H_27_O_3_ 327.1960; found, 327.1945.

IR– (ATR, neat, cm1): 3360 (br), 2955 (m), 2927 (m), 2855 (m), 1728 (m), 1713 (m), 1619 (s), 1360 (m), 1156 (m), 1059 (m), 836 (m).

#### Expression and Purification of CYPs

In the current manuscript, we have used three Cytochrome P450s, CYP3A4, CYP2C9, and CYP2D6. We expressed and purified both 2C9 and 3A4 as described previously The CYP2D6 construct was a gracious donation of Eric Johnson. The construct of CYP2D6 was also in the pcWori vector with modified and truncated N-terminal residues of MAKKTSSKGKL. We mention the procedures briefly here.

The CYP3A4 gene was in an NF-14 construct in the pcWori+ vector with ampicillin resistance and a C-terminal polyhistidine tag (Guengerich construct). The primary growth was done in Luria Bertini (LB) broth supplemented with Ampicillin (100 μg/mL). The secondary growth of the DH5alpha cells with the CYP3A4 gene was done in a total of 3 L in Terrific Broth media. The growth medium was supplemented with 0.1 M KPi buffer, 1 mM thiamine, ampicillin, and trace minerals. After the O.D. reached 0.4, we supplemented the growth media with arabinose and δ-ALA. The final expression was induced by adding 1 mM IPTG once the O.D. had reached between 0.8 and 1.0 in all the flasks. The protein growth was done for 48 h, and the cells were harvested by spinning at 8000 rpm for 15 min at 4 °C and stored at −80 °C. At the start of the purification process, the pellet was dissolved in a lysis buffer containing 20% glycerol (v/v), 0.1 M KPi, 500 mM Sodium Acetate and 5 mM and Beta mercaptoethanol (βME). We used 1 mM PMSF to inhibit proteases and sonicated the dissolved suspension on ice for a minimum of 4 cycles (60 s ON and 60 s OFF). The amplitude was set at 60%. The sonicated lysate was then subjected to ultracentrifugation at 30,000 rpm at 4 °C for 37 min. This step isolated the membrane fraction pellet, which was further dissolved in a second lysis buffer supplemented freshly with 1% cholate as a detergent. Once homogenized, the suspension was again subjected to sonication and ultracentrifuged using the same parameters mentioned above. Finally, the supernatant was loaded onto a Ni-NTA column and purified using a washing step supplemented with 15 mM imidazole, and a subsequent elution was done using 300 mM imidazole. The eluted protein was then further concentrated using 30 kDa amicons and buffer exchanged, removing imidazole.

CYP2C9 was also purified by similar methods with some modifications. Briefly, the protein growth and expression were done at 29 °C, 190 rpm for 48 h, and the cells were harvested at 4000g for 15 min. The pellet was resuspended in a suspension buffer (300 mL) consisting of 20% glycerol (v/v), 0.02 M KPi, 10 mM and β-mercaptoethanol (βME) and 1 mM PMSF. Once the pellet was completely resuspended, Lysozyme (0.3 mg/mL) was added to the buffer and further stirred for another 30 min at 4 °C. Spheroplasts were centrifuged at 5460 g for 30 min in the next step. The spheroplasts were stored at −80 °C before further processing. The spheroplasts were resuspended in 150 mL of buffer consisting of 500 KPi, 20% (v/v) Glycerol, 10 mM BME, 0.5 mM PMSF, Deoxyribonuclease (10 μg/mL), Ribonuclease A (10 μg/mL) and 0.1 mM Ibuprofen followed by sonication (40 s ON and 60 s OFF) for 6 cycles. The sonicated lysate was stirred at 4 °C for 90 min and then ultracentrifuged at 26000 rpm for 1 h. The composition of the column buffer used for equilibrating the Ni-NTA column and subsequent purification steps was 100 mM Potassium Phosphate, 20% (v/v) glycerol, 0.5% CHAPS and 1 mM BME. The washing step was done using the column buffer with the addition of 100 mM NaCl, 0.5 mM PMSF and 5 mM Histidine. The final elution was done using 10 mM KPi, 20% (v/v) glycerol, 0.5% CHAPS, one mM BME, 100 mM NaCl, 0.5 mM PMSF, and 50 mM Histidine (30 mL).

CYP2D6 was also purified by similar methods with some modifications. Briefly, the protein growth and expression were done at 30 °C, 190 rpm for 48 h, and the cells were harvested at 8000 rpm for 15 min. The pellet was resuspended in a Lysozyme buffer (300 mL) consisting of Tris HCl, pH 8, Sucrose (0.25 M), EDTA (0.25 mM) and freshly added Lysozyme (0.02 mg/mL). We pelted the spheroplast at 4000 rpm for 30 min. The spheroplasts were then resuspended in another buffer. The buffer composition included Glycerol (20%), 0.5 M KPi, 6 mM MgCl2, 5 mM βME, 0.2 mM PMSF, 1% Cholate and 0.1 mM Thioridazine followed by sonication (40 s ON and 40 s OFF) for 6 cycles. The sonicated lysate was then ultracentrifuged at 30000 rpm for 45 min. The composition of the column buffer used for equilibrating the Ni-NTA column and subsequent purification steps was 100 mM Potassium Phosphate, 20% (v/v) glycerol, 0.1% Cholate, 0.5 mM βME and 0.05 mM Thioridazine. The first washing step was done using the column buffer, adding 5 mM ATP, 10 mM MgCl2 and 150 mM KCl to remove the Gro complex. The second washing step used the column buffer base with 0.04 M l-histidine. The final elution was done using 0.1 M l-Histidine and 1 M NaCl in an elution volume of 100 mL.

#### Preparation of CYP Nanodiscs

Once the CYP proteins were purified, we aimed to incorporate them into lipid nanodiscs. The composition of the lipid nanodiscs included 100% POPC with purified membrane scaffold protein (MSP). We optimized the lipid-to-MSP ratio to 1:140 for the preparation of the nanodiscs. The method is described briefly here. The commercially available lipids were solubilized in CHCl_3_ to the required mol % and stored at −80 °C. To prepare the discs, we aliquoted out the required amount in 18 mm x100 mm test tubes and dried the lipids down consecutively under a stream of nitrogen. Once dried, the test tubes were stored in a desiccator overnight in a vacuum. The following morning, we solubilized the lipids using 200 mM cholate solution, warming the tubes in warm water. This step was followed by the sonication of the tubes for 15 min. After the sonication, we added everything, including MSP, glycerol, water and 0.1 M KPi and incubated the mixture for 45 min in a gentle rocker at 4 °C. Following the incubation, we added the calculated amount of CYP 30 and let it incubate for 45 min more. The Biobeads were added after the incubation and gently rocked at 4 °C for 7 h for CYP2C9 and CYP3A4. The incubation with biobeads was close to 12 h with CYP2D6. The more prolonged incubation with the Biobeads ensured the complete removal of the detergents. Once the incubation was over, we washed the Biobeads with 0.1 M KPi and filtered the mixture by spinning at 3000 rpm for 5 min. The eluent was concentrated down to the required amounts. The final concentrated nanodisc preparation was purified using size-exclusion chromatography using the AKTA Go FPLC with a Superdex 200 10/300 GL column in 0.1 M KPi, pH 7.4. The methods and the lipid ratio have been used for other CYPs.^53^

#### Expression and Purification of CYP 450 Reductase

The expression and purification of CYP450 reductase (CPR) were done using the previously described method.^6^

#### CYP-Mediated CBN Metabolism

We carried out concentration-dependent metabolism of CBN in the presence of 2 CYPs: 2C9 and 2D6. For the experiment, we used an enzyme concentration of 0.5 μM, reconstituted in the lipid system of POPC: POPS (8:2 v/v), and CPR at 0.6 μM dissolved in 0.1 M potassium phosphate buffer for CYP2C9. The substrate CBN was used in a range of 5–80 μM. The reaction mixture was incubated at 37 °C for 10 min, and then NADPH was added (1 mM), allowing the reaction to proceed for an additional 30 min. We quenched the reactions using a mixture of Hexane: Ethyl Acetate (8:2 v/v) and used the same composition to extract the CBN metabolites. We did a total extraction for three cycles. Following the extractions, the extractions were dried under a continuous stream of nitrogen, and the residual products were finally dissolved in 90% ethanol. We used CYP2D6 in a nanodisc for the metabolism of CBN. Since the protein was in a nanodisc, we did not add the lipid mixture separately, but the rest of the components were the same. We carried out the same protocol for the metabolism of CBN using 1 μg of human liver microsome, CPR (0.6 μM) and NADPH (1 mM).

#### CBN Administration in Female Mice

Initially dissolved in ethanol, CBN was combined with a volume of Tween 80, constituting 6% of the total solution volume for injection. Ethanol was eliminated via vacuum evaporation, and saline was added to prepare a 4 mg/mL solution. CBN (20 mg/kg) or vehicle was administered to C57BL/6 mice intravenously (IV) through the retro-orbital sinus or intraperitoneally (IP) at the lower right quadrant of the abdomen. Animals were euthanized at 30- or 120 min postinjection. Blood samples, collected via cardiac puncture into heparin tubes, underwent cold centrifugation at 4500 rpm for 15 min to isolate plasma. Plasma and whole liver samples were flash-frozen at −80 °C for subsequent analysis. Our decision to use female mice for this experiment was based on the availability of age-matched mice in the colony at the time of the experiment.

Studies utilizing mice were performed under a protocol approved by the University of California, San Francisco’s Institutional Animal Care and Use Committee (IACUC) and in accordance with the ACS Ethical Guidelines for Publication of Chemical Research (Ethics Approval Number: AN199674-00C and AN200700-00).

#### Retro-Orbital Injection Procedure

Mice were anaesthetized with a 3% inhalant dose of isoflurane until their respiratory rate decreased to 50% of baseline (i.e., 40–60 breaths/min) and the righting reflex was abolished. Mice were subsequently removed from the induction chamber and laid on their side. Using the index finger and thumb of the nondominant hand, the skin above and below the eye was retracted until a slight protrusion from the socket was noted. A 29G1/2 needle was inserted, bevel down, into the medial canthus at an approximate 45-degree angle until slight resistance was noted. Volume was slowly infused at a rate of 15uL/second. The needle was subsequently removed from the eye socket, and ophthalmic ointment was added.

#### Intraperitoneal Injection Procedure

Animals were gently restrained by scruff, and the injection site at the lower right quadrant of the abdomen was identified. The needle was inserted at a 60-degree angle to the body, and the plunger was lightly withdrawn to ensure the needle was not in contact with viscera or organs before dispensing volume.

#### Extraction of CBN and CBN Metabolites from Plasma Samples

200 μL plasma samples were collected and used for extraction using 1 mL of Hexane: Ethyl acetate (8:2 v/v) following a similar protocol described above.

#### LC/MS/MS Analysis of Extractions from Plasma Samples

Samples were analyzed using the Sciex QTrap 6500+ system (Framingham, MA) with Waters Acquity I- class plus UPLC. Software Analyst 1.7.1 was used for data acquisition.

The LC separation was performed on a Waters Acquity UPLC BEH column (2.1 × 100 mm, 1.7 μm) with mobile phase A (0.1% formic acid in water) and mobile phase B (0.1% formic acid in acetonitrile). The flow rate was 0.5 mL/min unless noted otherwise. The linear gradient was as follows: 0–0.5 min, 70%A; 2 min, 20%A; 4–4.2 min, 0%A; 4.21–5.2 min, 0%A (0.8 mL/min); 5.3–6.9 min, 70%A. The autosampler was set at 10 °C, and the column was kept at 50 °C. The injection volume was 5 μL.

Mass spectra were acquired under positive electrospray ionization (ESI) with the ion spray voltage of 4500 V. The source temperature was 500 °C. The curtain gas, ion source gas 1, and ion source gas 2 were 32, 50, and 70 psi, respectively. Multiple reaction monitoring (MRM) was used for quantitation with the MRM transition of CBN (*m*/*z* 311.2 → *m*/*z* 269.1), CBN-1′–OH (*m*/*z* 327.2 → *m*/*z* 253.1), CBN-11-OH (*m*/*z* 327.2 → *m*/*z* 128.1), CBN-*p-*quinone (*m*/*z* 325.2 → *m*/*z* 283.1) and EPEA-d4 as internal standard (*m*/*z* 350.3 → *m*/*z* 66.1).

#### Quantitation of CBN Metabolites by LC/MS-MS

Samples were analyzed with the Sciex QTrap 6500+ system (Framingham, MA) with Waters Acquity I-class plus UPLC. Software Analyst 1.7.1 was used for data acquisition.

The LC separation was performed on a Waters Acquity UPLC BEH column (2.1 × 100 mm, 1.7 μM) with mobile phase A (0.1% formic acid in water) and mobile phase B (0.1% formic acid in acetonitrile). The flow rate was 0.5 mL/min unless noted otherwise. The linear gradient was as follows: 0–0.5 min, 70%A; 2 min, 20%A; 4–4.2 min, 0%A; 4.21–5.2 min, 0%A (0.8 mL/min); 5.3–6.9 min, 70%A. The autosampler was set at 10 °C, and the column was kept at 50 °C. The injection volume was 5 μL.

Mass spectra were acquired under positive electrospray ionization (ESI) with the ion spray voltage of 4500 V. The source temperature was 500 °C. The curtain gas, ion source gas 1, and ion source gas 2 were 32, 50, and 70 psi, respectively. Multiple reaction monitoring (MRM) was used for quantitation, as described in transitions CBN (*m*/*z* 311.2 → *m*/*z* 205.1), CBN-1′–OH (*m*/*z* 327.2 → *m*/*z* 253.1), CBN-11-OH (*m*/*z* 327.2 → *m*/*z* 291.1), CBN-*p-*quinone (*m*/*z* 325.2 → *m*/*z* 283.1) and CBC-d9 as internal standard (*m*/*z* 324.2→ *m*/*z* 202.1).

#### Data Analysis

All data were analyzed using the software Skyline (daily version 22.2.1.278). It includes raw data import, peak integration, and a linear regression fit with 1/×2 weighting for the calibration curves. QCs and standards have good signals and linear responses in the calibrated concentration ranges.

#### Untargeted Mass Spectrometry (LC/MS) Methods

The untargeted mass spectrometry to determine the primary metabolite products were done using an Acquity UPLC BEH with a C18 column (100 mm × 2.1 mm, 1.7 μm). Mobile A phase consists of water and 0.1% formic acid, and mobile phase B consist of 20% acetonitrile (ACN), 80% isopropyl alcohol (IPA) and 0.1% formic acid. The column temperature was 60 °C, and the injection volume was 2 μL. The scan range for mass spectrometry was 150–2000 *m*/*z*. The analysis of raw files was done using the Thermo Fisher Xcalibur software.

#### Biological Study of CBN and Its Metabolites on BV2 Microglial Cells

The initial goal was to investigate the relative cytotoxicity of the molecules. To do this, we performed a Lactate Dehydrogenase assay (LDH) assay, monitoring the LDH release into the medium upon treatment of the metabolites at a concentration range between 1 and 10 μM. The assay was performed using the LDH Assay kit from Cayman Chemicals. The BV2 microglial cells were cultured in DMEM supplemented with FBS and 1× P/S. 50,000 cells were plated in a 96-well plate and grown for 24 h. After 24 h, the cells were treated with various molecules in triplicate across the specified concentration range. The assay was performed 24 h after the cells were treated with the molecules.

#### Determination of Nitric Oxide (NO) Levels and LDH Levels

The BV2 microglial cells were subjected to the treatment of LPS both in the absence and presence of CBN and its selected metabolites. The seeding density for this assay was 1 × 10^5^ cells/well. The cells were initially treated with the molecules for 4 h before being stimulated with LPS (25 ng/mL). The NO assay was performed 24 h after the treatment with LPS. The NO assay was performed according to the manufacturer’s protocol, and NO levels were quantified using nitrate standard curves. For the assay, 50 μL of cell media was used in a 96-well plate, and the absorbance was recorded at 540 nm. All measurements were performed in *n* = 5.

Following the manufacturer’s protocol, the same supernatant was used to assess LDH levels using the LDH assay kit.

#### Proinflammatory and Anti-inflammatory Expression Markers Using Enzyme-Linked Immunosorbent Assays (ELISA)

ELISA assays of IL-6, IL-10 (Item No. 88–7105), and Arginase 1 were performed using the same supernatant with which we had completed the NO and LDH assays. The culture media was collected, and the IL-6 (Thermo Scientific) and Arginase 1 (abcam) assays were performed using the specific monoclonal antibodies as per the manufacturer’s instructions.

#### Reagents and Plasmids for GPCR Study

We purchased CP-55,940 from Cayman Chemicals (Item No. 13241). Codon-optimized sequences encoding for CB1R, CB2R, GPR55, and GPR119 were a kind gift from Dr. Bryan Roth (University of North Carolina, NC) (Add gene, PRESTO-Tango Kit- #1000000068). GPCR coding sequences were subcloned by In-Fusion HD Cloning (Clontech) in a pcDNA3.1 vector, removing the V2-tail, TEV domain, and tTA sequences. All constructs were verified by Sanger sequencing.

#### Cell Culture and Transfection

We purchased HEK293*T*/17 cells (RRID: CVCL_1926) from ATCC. The cells were cultured in DMEM (Gibco, 10567-014) supplemented with 10% FBS dialyzed (Biowest, S181D), nonessential amino acids (Gibco, 11140-050), penicillin 100 units/mL and streptomycin 100 μg/mL (Gibco, 15140-122), and amphotericin B 250 μg/mL (Thermo Fisher, 15290-018) at 37 °C and 5% CO_2_. The cells were routinely monitored for possible mycoplasma contamination. We seeded two million cells in each well of 6-well plates in medium without antibiotics for 4 h and then transfected with a 1:3 ratio of total DNA plasmids (2.5 μg) and polyethylenimine (PEI; 7.5 μL) (Polysciences, 23966). We kept a 4:1 ratio between transfected Gα proteins and Gβγ proteins. Accordingly, the following amount of each DNA plasmid was used for transfection: 13 ng of masGRK3CT-Nluc, 208 ng of β1-Venus (156-239), 208 ng of γ2-Venus (1-155), 833 ng for each Gα protein, 208 ng of receptor, 208 ng for chaperones RIC8B (for Gs) or RIC8A (for Gq, G11). The empty vector pcDNA3.1 was used to normalize the ratio of transfected plasmids. We transiently transfected cells and incubated them for 16 h. Then, we starved cells in Optimem (Gibco, #11-058-021) for 6 h before being tested.

#### G Protein Nano BRET Assay

We collected the serum-starved cells in 1.5 mL tubes and spun them at 500g for 5 min at room temperature. The cell pellet was resuspended in 250 μL of BRET buffer (PBS supplemented with 0.5 mM MgCl_2_ and 0.1% glucose). Thirty μL cells were plated in each well of white 96-well microplates (Greiner Bio-One). The nanoluc substrate used in the experiments is NanoGlo (Promega, N1120), diluted 1:250 in BRET buffer, and 30 μL was applied to plated cells. The BRET measurements were obtained using a POLARstar Omega microplate reader (BMG Labtech). All the measurements were obtained at room temperature. The BRET signal was determined as the ratio of the light emitted by Gβ1γ2-venus (emission filter 535/30) to the light emitted by masGRK3CT-Nluc (475/30). For (Figure 8A,B) experiments, NanoBRET procedures were performed as previously.^32^ Briefly, all cells were plated and mixed with NanoGlo. Initial readings were used to establish a basal BRET ratio, and then 60 μL of 2x agonist was added. Emission intensity was recorded for 5 min. ΔBRET was obtained by subtracting the basal BRET ratio from the maximal BRET ratio obtained after vehicle/agonist application. The ΔBRET obtained on pcDNA3.1 (empty vector) transfected cells was subtracted from the receptor conditions.

For the antagonist mode (Figure 8C,D), 30 μL of cells were combined with 30 μL of NanoGlo (1:250) and read for a baseline of 2 min. Then, we added 60 μL of 2× agonist (CP 55,940) (final concentration 1 μM), and we recorded until the BRET ratio reached a plateau. Then, we applied 60 μL of vehicle, CBN, and the metabolites from 0.01 to 50 μM. Measurements continued for 100 s. ΔBRET is obtained, as previously mentioned, and normalized to 100% of the maximal BRET ratio obtained upon agonist application.

#### Molecular Docking and Molecular Dynamics (MD) Simulations

We obtained the full-length CYP2C9 structure from the AlphaFold Protein Structure Database^54^ and determined its membrane orientation using the Orientations of Proteins in Membranes (OPM).^55^ The OPM-oriented structure was loaded into the CHARMM-GUI Membrane to construct a membrane-bound system embedding CYP2C9 in a bilayer composed entirely of 1-palmitoyl-2-oleoylphosphatidylcholine (POPC), which represents the primary lipid type of the endoplasmic reticulum membrane. The protein termini were capped with an acetylated amino terminus (ACE) and an *N*-methylamide carboxy terminus (CT3). We integrated the missing heme group from the CYP2C9 crystal structure (PDB ID: 1R9O), along with the associated water molecule coordinating the heme, using the PSFGEN plugin of Visual Molecular Dynamics.^56^ Protonation states of key residues were estimated using p*K*_a_ calculations in Maestro’s PropKa tool^57^ and modeled via PSFGEN. The system was solvated with the SOLVATE plugin and neutralized with 0.15 M NaCl using VMD’s AUTOIONIZE plugin. Subsequently, the membrane-bound CYP2C9 system was simulated for 1000 ns, with further details on simulation parameters provided below.

#### Docking of Cannabinoids to CYP2C9

To characterize the putative binding modes of cannabinoids (CBN, THC) to CYP2C9, we performed molecular induced-fit docking using the Molecular Operating Environment (MOE) software (2024.06, Chemical Computing Group ULC, Montreal, QC). We used the CYP2C9 structure from PDB ID: 1R9O and completed missing segments (the N-terminal transmembrane helix, residues 38-VIGNI-42, and 214-QICNNFS-220) using MOE’s homology modeling tool. To guide this, we aligned a snapshot from the end of our membrane-bound CYP2C9 simulation with the PDB structure (1R9O). In the homology model tool, we selected 1R9O as the template structure, specified the membrane-bound CYP2C9 regions to override the missing regions, and set the environment to include the ligand and water molecules. These settings retained most of the protein from the 1R9O crystal structure while reconstructing only the missing sections using the AlphaFold model incorporated in our membrane-bound system.

Cannabinoid structures were obtained from the PubChem database^58^ and prepared in MOE using the ‘conformation import’ workflow to generate multiple low-energy conformations. These conformations were docked to the active site of CYP2C9, defined by the location of the crystal ligand, flurbiprofen in PDB: 1R9O. For induced-fit docking in MOE, we used the following settings: the placement method was ‘triangle matcher,’ the refinement method was ‘induced fit’ (targeting residues within 6 Å of the cannabinoid), and the scoring function was GBVI/WSA dG with the AMBER10 force field. We saved the top 20 docking poses for each cannabinoid. The GBVI/WSA dG scoring function was selected as it was the only one able to capture the crystal position of flurbiprofen accurately. A summary of the docking scores for each cannabinoid is presented in Table S1. Since the top 20 docked poses of each cannabinoid displayed minimal variation in docking scores (Table S1), we applied additional metrics to further explain the Results section to select the best pose for subsequent simulations. The hydrogen bond occupancy of CBN and THC is shown in Table S2.

#### MD Simulation of Cannabinoid-Bound CYP2C9

The selected CYP2C9-cannabinoid complexes were embedded in a pure POPC membrane using the final frame of the previously simulated membrane-bound CYP2C9 AlphaFold model. The systems were then solvated with VMD’s SOLVATE plugin and neutralized to 0.15 M NaCl using the AUTOIONIZE plugin. Each membrane-bound CYP2C9 system, with its docked cannabinoid, was subsequently simulated for 1000 ns, with additional details of the simulation protocol provided below.

The interaction probability of residues with cannabinoids was calculated using a distance metric to define an interaction. Specifically, an interaction was defined as any heavy atom of a residue being within 4 Å of any heavy atom of a cannabinoid. The number of interacting residues was counted at each nanosecond of the simulation, and the total interactions for each residue were normalized by the total simulation time of 1000 ns. Thus, an interaction probability of 1.0, as shown in Figure S8E,F, indicates that the residue remained within 4 Å of the cannabinoid throughout the entire simulation. Interaction energies between key interacting residues and each cannabinoid were calculated throughout the simulations using the NAMD Energy plugin in VMD.

To estimate binding free energies, we employed the Prime MM-GBSA module in Schrödinger’s Maestro (Schrödinger Release 2024-4: Maestro, Schrödinger, LLC, New York, NY, 2024.), using the VSGB solvation model and OPLS4 force field. Residues within 4 Å of the ligand were treated as flexible, with the sampling method set to ‘minimize.’

#### MD Simulation Details

The membrane-bound CYP2C9 AlphaFold model, along with each CYP2C9-cannabinoid complex, was simulated using NAMD3 ^59,60^ with CHARMM36m^61^ parameters for the protein, CHARMM36 ^62^ parameters for lipids, and the TIP3P water model,^63^ applying a 2 fs time step. Cannabinoid parameters were obtained from the CHARMM General Force Field (CgenFF) Web server.^64^ Each system underwent energy minimization for 10,000 steps, followed by a four-step equilibration process with gradually relaxed restraints to facilitate the cannabinoids’ accommodation within the CYP2C9 active site.

During equilibration, lipid tails were initially allowed to melt while everything except waters and ions were restrained with a 1 kcal mol^–1^Å^–2^ force constant for 1 ns. Then, all lipid atoms, waters and ions were allowed to move while protein, heme, ligand, and heme-coordinated water oxygens were restrained with a 1 kcal mol^1^ å^2^ force constant for 2 ns. Subsequently, protein backbone, heme, and cannabinoid-heavy atoms were restrained at 0.5 kcal mol^–1^Å^–2^ for an additional 2 ns. Finally, Cα atoms and cannabinoid ring systems were restrained with force constants of 0.5 and 0.2 kcal mol^–1^Å^–2^, respectively, for 4 ns. Each equilibrated system was then subjected to a 1000 ns production simulation without restraints. The temperature was maintained at 310 K using the Langevin thermostat,^65^ and pressure at 1 bar using the Nosé-Hoover piston method.^66^ Long-range electrostatics were calculated with the particle mesh Ewald (PME) method^67^ using a 1 Å grid spacing, while nonbonded forces employed a 12 Å cutoff and a 10 Å switching distance. Simulations were visualized and analyzed using VMD.

#### Statistical Analyses

All statistical analyses have been done using GraphPad Prism version 10.2.3 for Windows, GraphPad Software, Boston, Massachusetts, USA. The results are represented as mean ± SEM. We used the one-way ANOVA followed by Dunnett’s multiple comparison tests to calculate statistical significance.

## Supplementary Material









## Notes

NMR, mass spectrometry and HPLC analysis of synthesized compounds, and other discussions with respect to binding data (PDF)
SMILES of all the major compounds in this paper, with their relevant data (CSV)
Homology modeling (PDB)
Template (PDB ID 1R9O) (PDB)

## Abbreviations

Ac_2_O: acetic anhydride
AIBN: azobis­(isobutyronitrile)
AgOAc: silver acetate
BME: beta mercaptoethanol
BRET: bio resonance energy transfer
CB1R: cannabinoid receptor 1
CB2R: cannabinoid receptor 2
CBC: cannabichromene
CBG: cannabigerol
CBN: cannabinol
CBN-11-OH: cannabinol-11-hydroxy
CBN-1′–OH: cannabinol-1′-hydroxy
CBNQ: cannabinol-p-quinone
CHAPS: 3-[(3-cholamidopropyl)­dimethylammonio]-1-propanesulfonate
COM: center of mass
CPR: cytochrome P450 reductase
CTB: cell-titer blue
CYP450: cytochrome P450
DCE: dichloroethane
DMAP: 4-dimethylaminopyridine
EDTA: ethylenediaminetetraacetic acid
ELISA: enzyme linked immunosorbent assay
FBS: fetal bovine serum
FPLC: fast protein liquid chromatography
HLM: human liver microsomes
IV: intravenous
IP: intraperitoneal
IL: interleukin
LC/MS: liquid chromatography mass spectrometry
LDH: lactate dehydrogenase
LPS: lipopolysaccharide
MeOH: methanol
MSP: membrane scaffold protein
MRSA: methicillin-resistant
MD: molecular dynamics
MM-GBSA: molecular mechanics generalized born surface area
NADPH: nicotinamide dinucleotide phosphate hydrogen
NMR: nuclear magnetic resonance
NO: nitric oxide
PDB: protein data bank
PMSF: phenylmethanesulfonylfluoride
RMSD: root-mean-square division
SeO_2_: selenium dioxide
THC: tetrahydrocannabinol
TBHP: tert butyl hydroperoxide
TRP: transient receptor potential
UGT: UDP-glucuronosyltransferase;
