# Supplementary material for: Distinct Interactions of Cannabinol and Its Cytochrome P450-Generated Metabolites with Receptors and Sensory Neurons
Source: J Med Chem. 2025 Jun 26;68(13):13935–53. doi: 10.1021/acs.jmedchem.5c00938 (PMC12257535; doi:10.1021/acs.jmedchem.5c00938)
Supplement: Supplementary file 1 [file jm5c00938_si_001.pdf]

Supporting Information

Distinct Interactions of Cannabinol and Its Cytochrome P450-Generated Metabolites with Receptors and Sensory Neurons

Debanjan Kundu[†], Luca Franchini[°], Hale S Hasdemir[§], Elliot Lloyd[#], Jonathan Maturano[‡], Katalin Rabl[#], Anna Nicole Denissiouk[†], Mark Schumacher[#], David Sarlah[$], Judith Hellman[#], Emad Tajkhorshid[§], Cesare Orlandi[°] and Aditi Das[†]*

[†]School of Chemistry and Biochemistry, College of Sciences, Georgia Institute of Technology, IBB, Parker H. Petit Institute for Bioengineering and Biosciences, Atlanta, GA 30332.

◦Department of Pharmacology and Physiology, University of Rochester Medical Center, 601 Elmwood Ave, Rochester, NY 14642

[§]Theoretical and Computational Biophysics Group, NIH Resource for Macromolecular Modeling and Visualization, Beckman Institute for Advanced Science and Technology, Department of Biochemistry, and Center for Biophysics and Quantitative Biology, University of Illinois at Urbana−Champaign, Urbana, Illinois 61801, United States

[‡]Roger Adams Laboratory, Department of Chemistry, Cancer Center at Illinois, University of Illinois, Urbana, Illinois 61801, United States

[$]Department of Chemistry, Rice University, Houston, TX, 77005

[#]Department of Anesthesia and Perioperative Care, University of California San Francisco, San Francisco, CA, 94143

***** *Corresponding author: aditidas@chemistry.gatech.edu*

Table of Contents

1.1- 1H NMR and 13C NMR Spectra 3

1.2- HPLC traces of synthesised CBN-1′-OH and CBN p-quinone 8

Figure S1: Potential metabolites of Cannabinol (CBN) metabolism 10

Figure S2: Potential glucuronidated metabolites of Cannabinol (CBN) metabolism and CBN-11-OH 11

Figure S3: LC/MS of CBN and CBN-11-OH glucuronidation 12

2.0- Discussions on the binding of CBN and other substrates with CYP2D6, CYP3A4 Nanodisc binding experiments with CBN 12

Figure S4: Binding of Cannabinol and Bromocriptine with CYP3A4 in nanodisc 13

2.1-NADPH Oxidation Assays 13

Figure S5: NADPH Oxidation of CBN and BCT with CYP3A4 ND 14

2.2- CYP2D6 Nanodisc binding experiments with CBN 14

Figure S6: Binding of CBN with CYP2D6 in nanodisc 15

Figure S7: Effect of CBN and its metabolites (LDH and Arginase Assay) 16

Figure S8: *In silico* work on THC binding with CYP2C9 17

Figure S9: LC/MS/MS of CBN and CBN-11-OH standard molecules 18

1.1- 1H NMR and 13C NMR Spectra

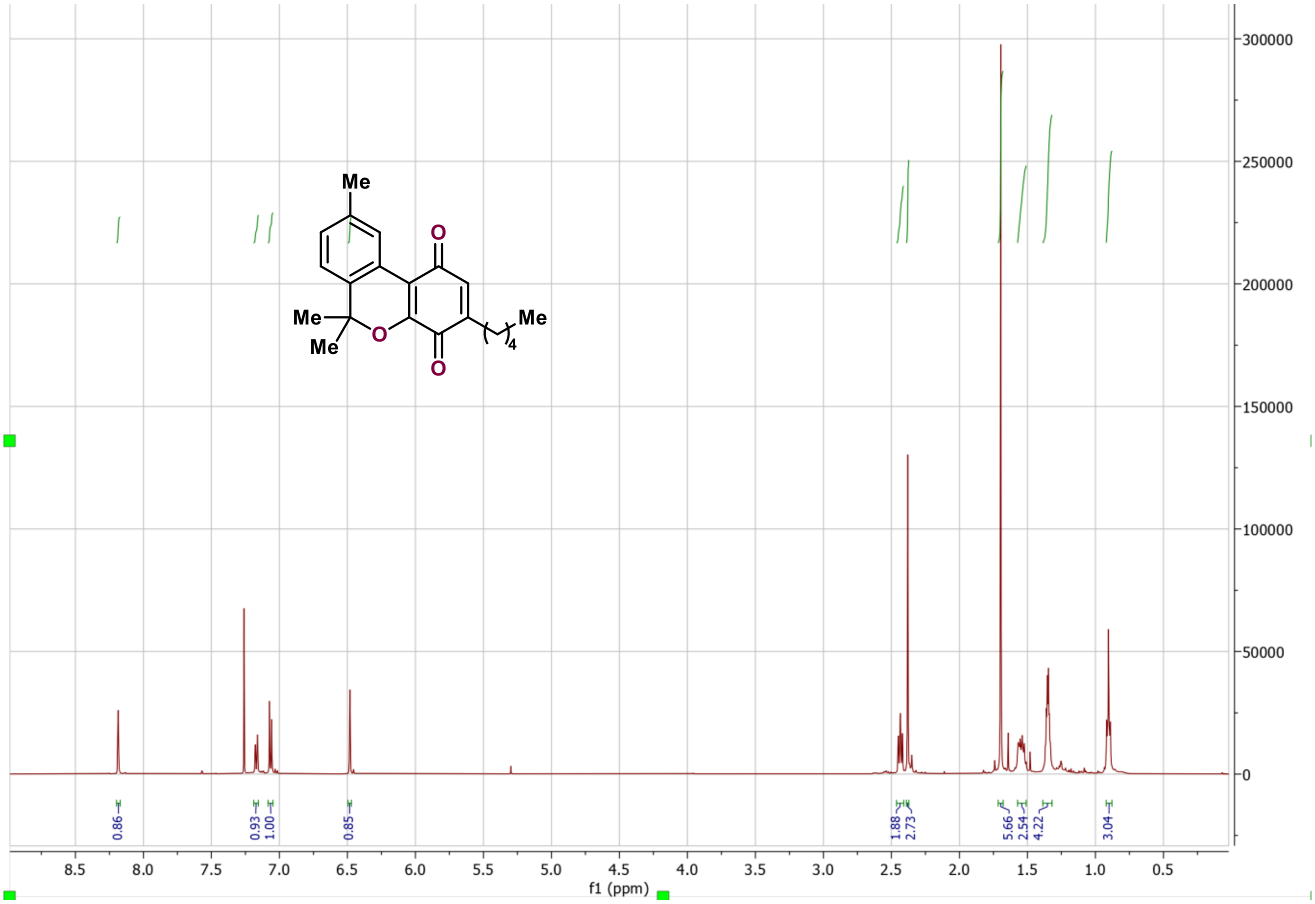

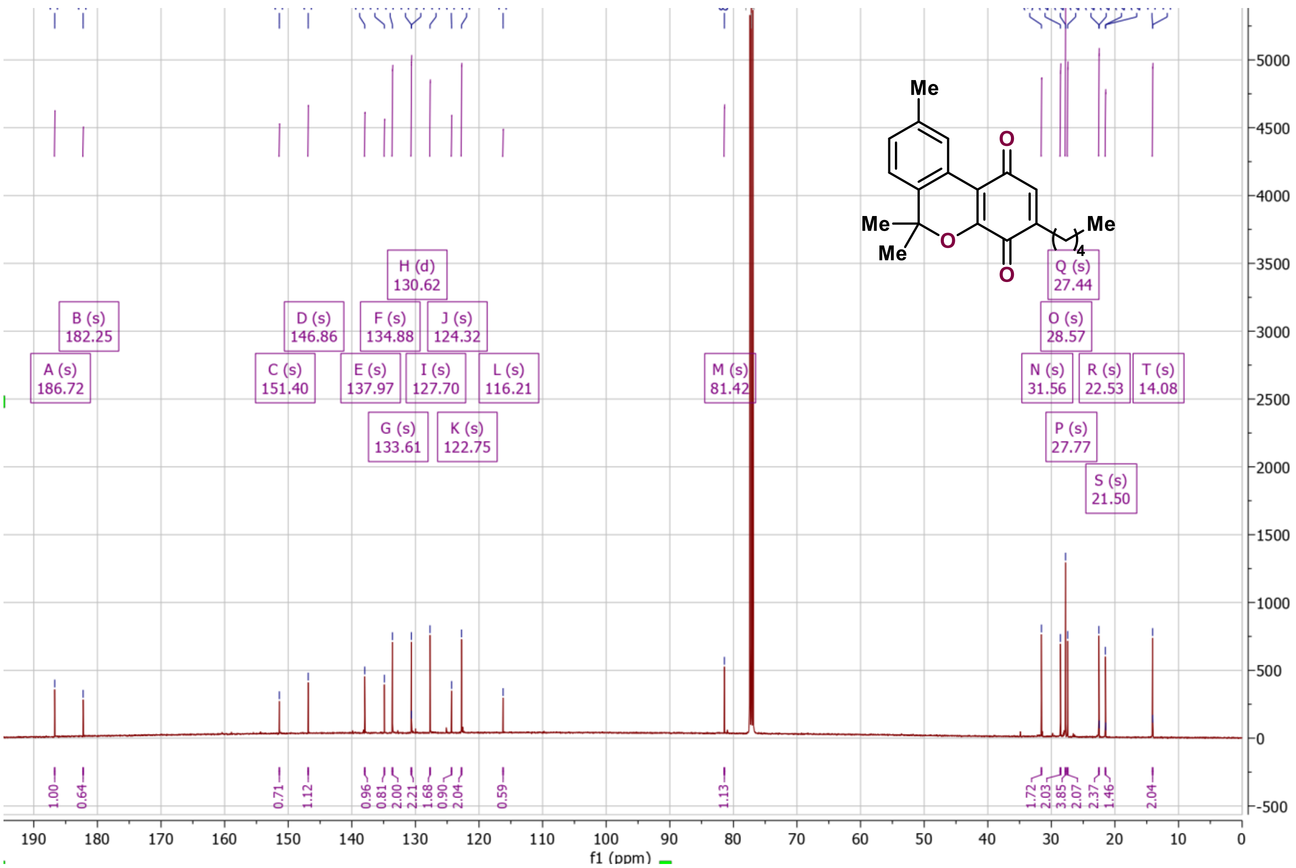
A (s) 186.72
B (s) 182.25
C (s) 151.40
D (s) 146.86
E (s) 137.97
F (s) 134.88
G (s) 133.61
H (d) 130.62
I (s) 127.70
J (s) 124.32
K (s) 122.75
L (s) 116.21
M (s) 81.42
N (s) 31.56
O (s) 28.57
P (s) 27.77
Q (s) 27.44
R (s) 22.53
S (s) 21.50
T (s) 14.08
Me
O
O
Me
Me
Me
O
4
f1 (ppm)

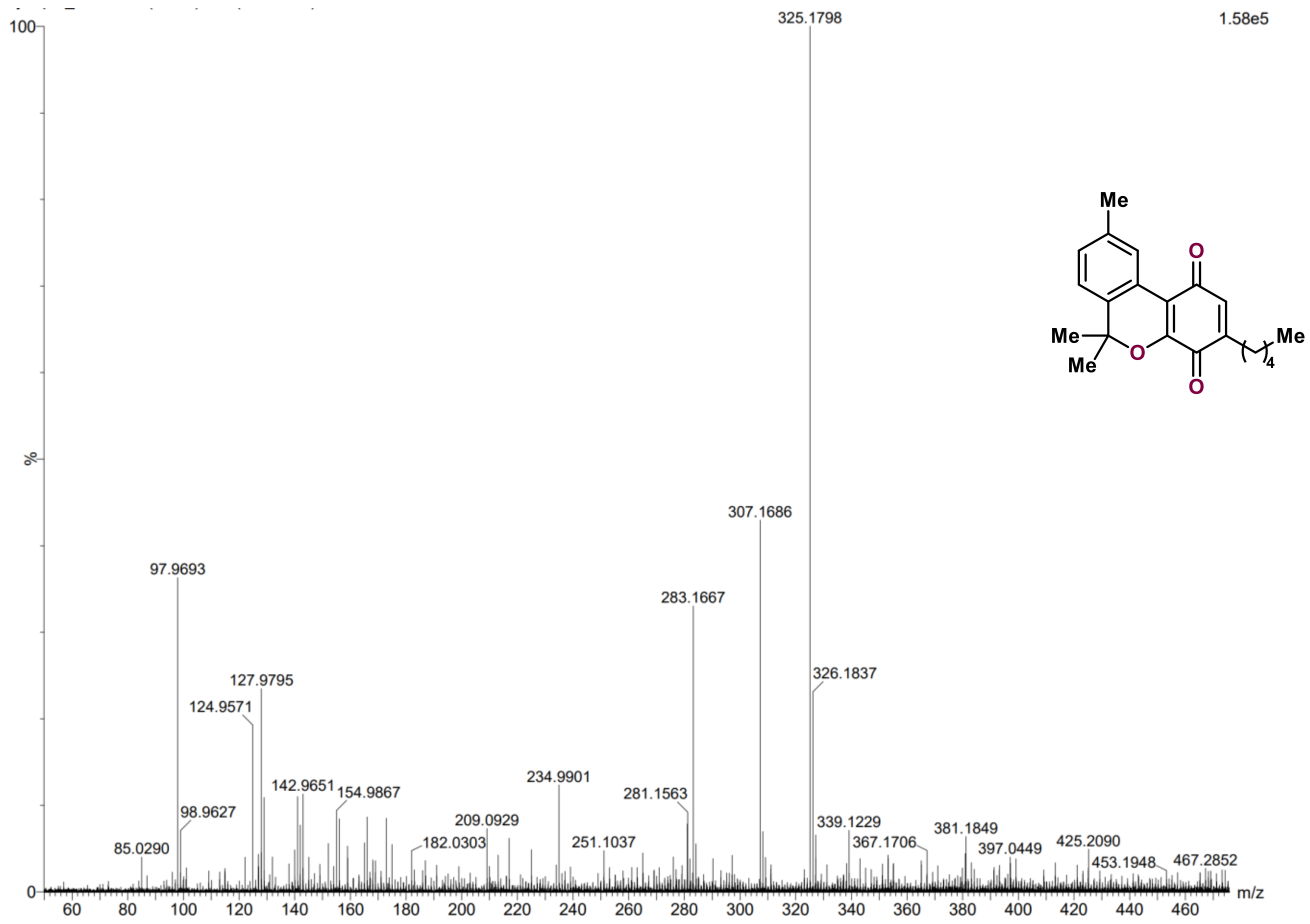
325.1798
1.58e5
100
%
0
307.1686
97.9693
283.1667
127.9795
124.9571
326.1837
142.9651
154.9867
234.9901
281.1563
98.9627
209.0929
339.1229
381.1849
85.0290
182.0303
251.1037
367.1706
425.2090
397.0449
467.2852
453.1948
m/z
Me
O
Me
Me
O
O
Me
4

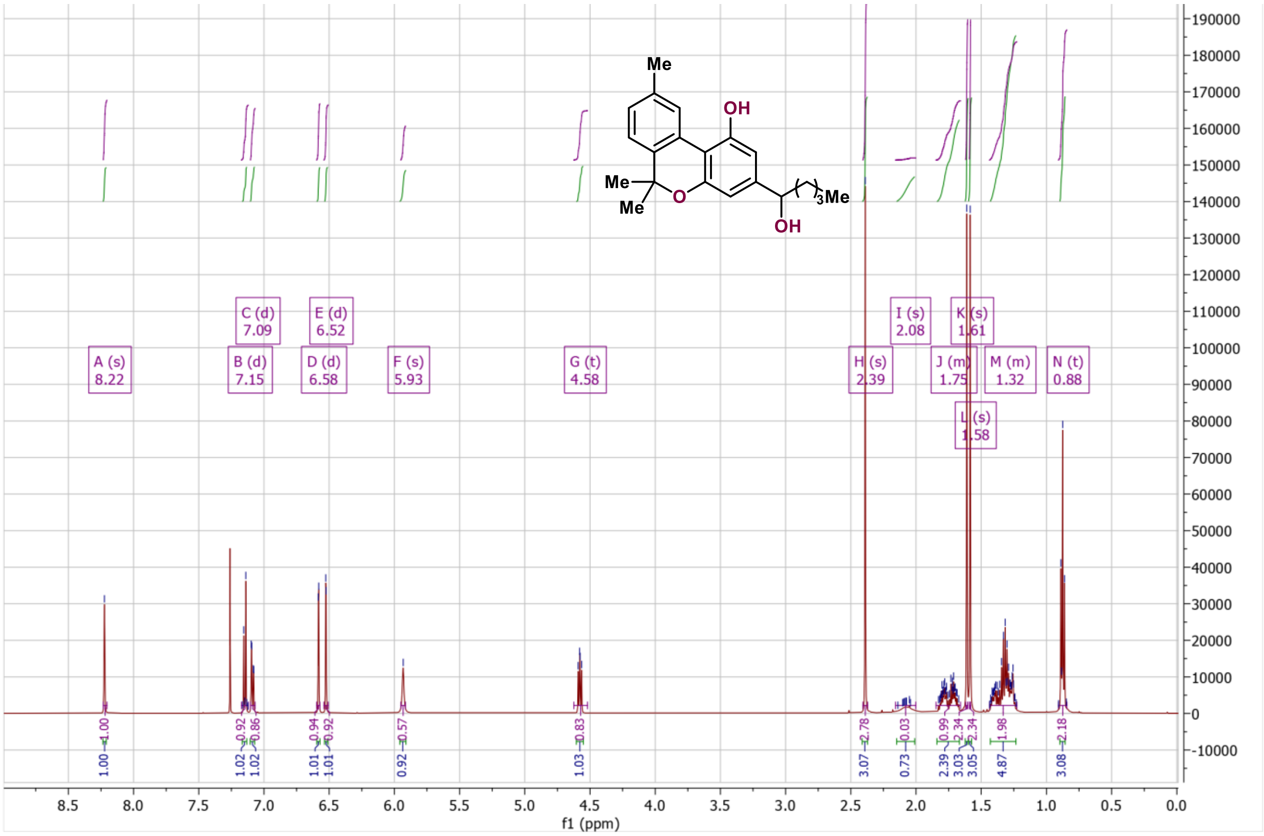
Me
OH
Me
Me
O
3Me
OH
A (s)
8.22
B (d)
7.15
C (d)
7.09
D (d)
6.58
E (d)
6.52
F (s)
5.93
G (t)
4.58
H (s)
2.39
I (s)
2.08
J (m)
1.75
K (s)
1.61
L (s)
1.58
M (m)
1.32
N (t)
0.88
f1 (ppm)

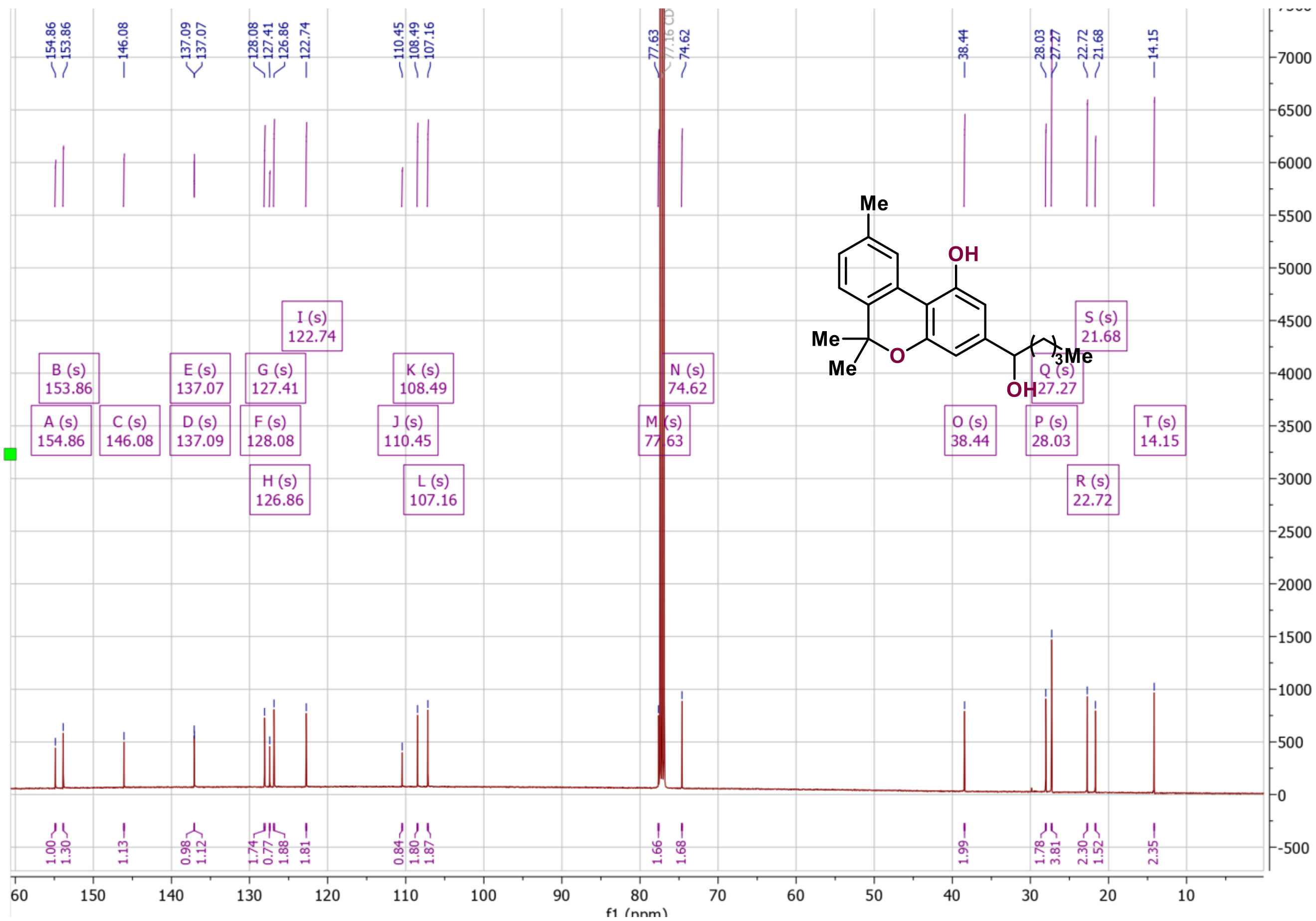

1.2- HPLC traces of synthesised CBN-1′-OH and CBN p-quinone

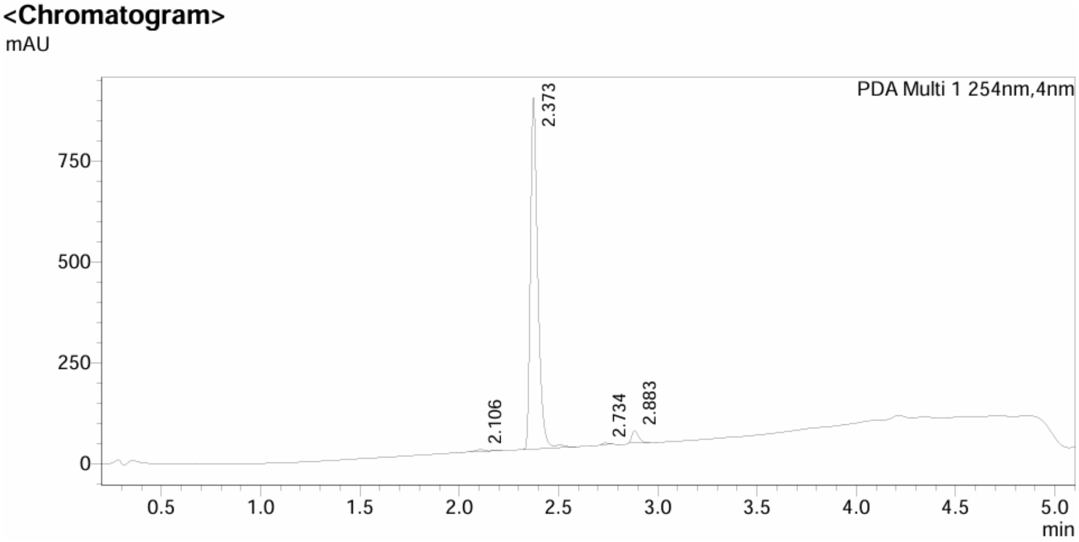

Peak Table

PDA Ch1 254nm

Peak#	Ret. Time	Area	Area%
1	2.106	15125	0.637
2	2.373	2282906	96.170
3	2.734	7464	0.314
4	2.883	68325	2.878
Total		2373821	100.000

HPLC trace of CBN-1′-OH showing greater than 95% purity.

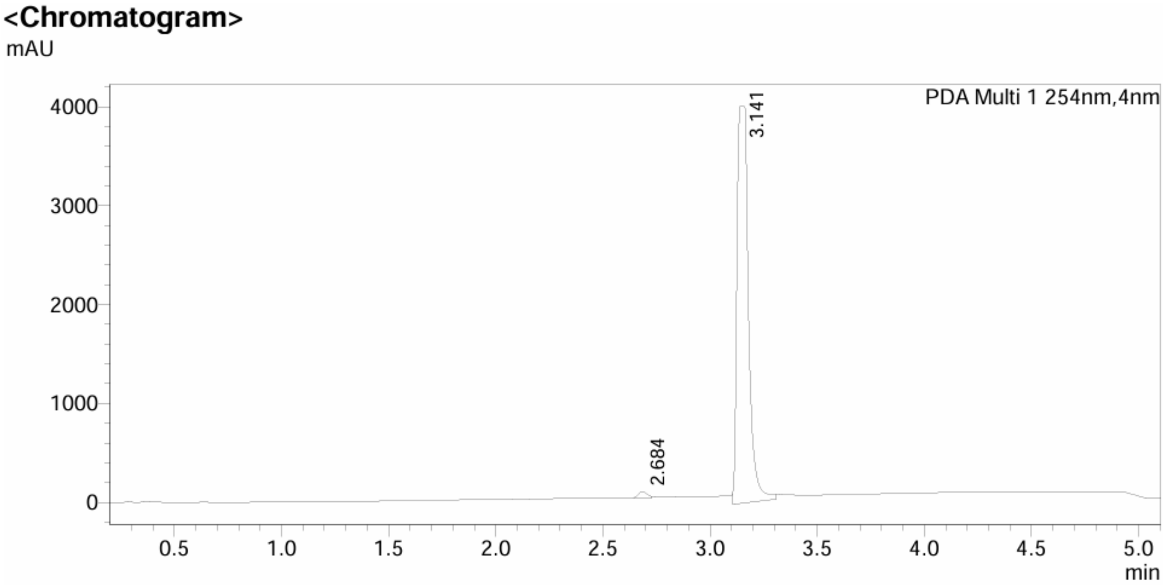

Peak Table

PDA Ch1 254nm

Peak#	Ret. Time	Area	Area%
1	2.684	162479	1.082
2	3.141	14849881	98.918
Total		15012360	100.000

HPLC trace of CBN *p*-quinone showing greater than 95% purity.

Figure S1: Potential metabolites of Cannabinol (CBN) metabolism

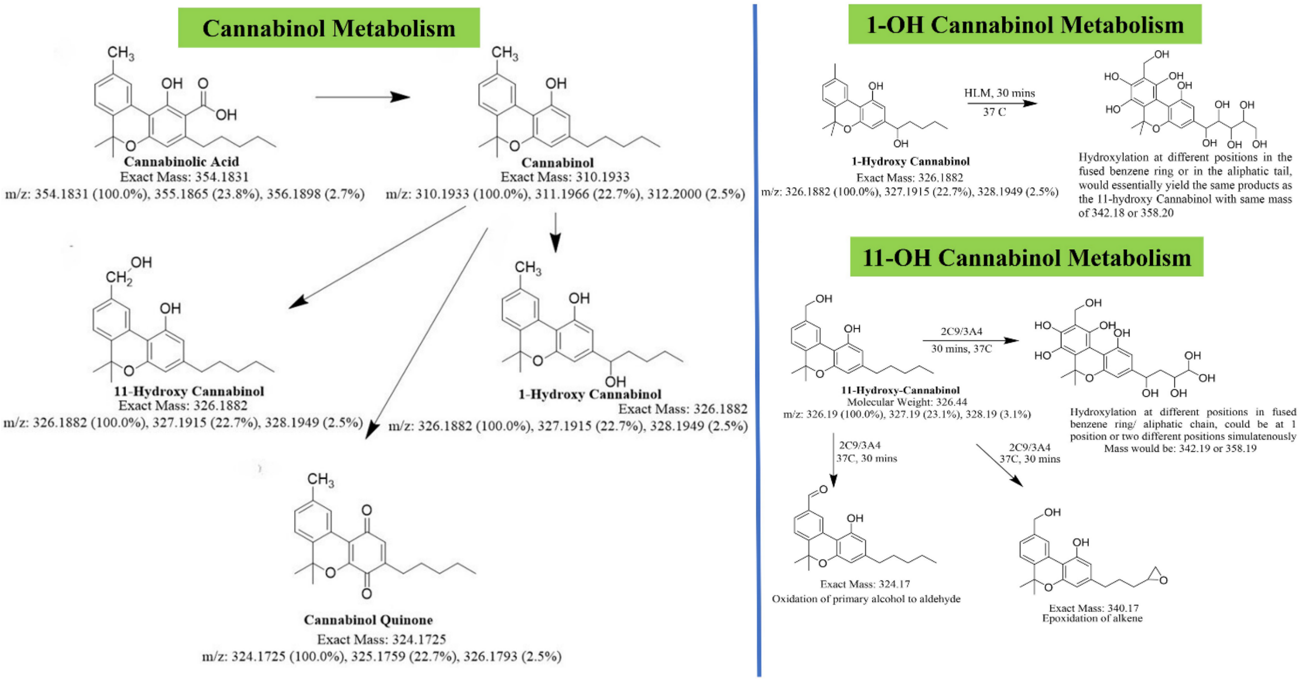

Figure S1: Chemdraw illustrations of various metabolites of Cannabinol and the respective metabolites of CBN-11-OH and, CBN-1'-OH as predicted by the Bio transformer 3.0 software

Figure S2: Potential glucuronidated metabolites of Cannabinol (CBN) metabolism and CBN-11-OH

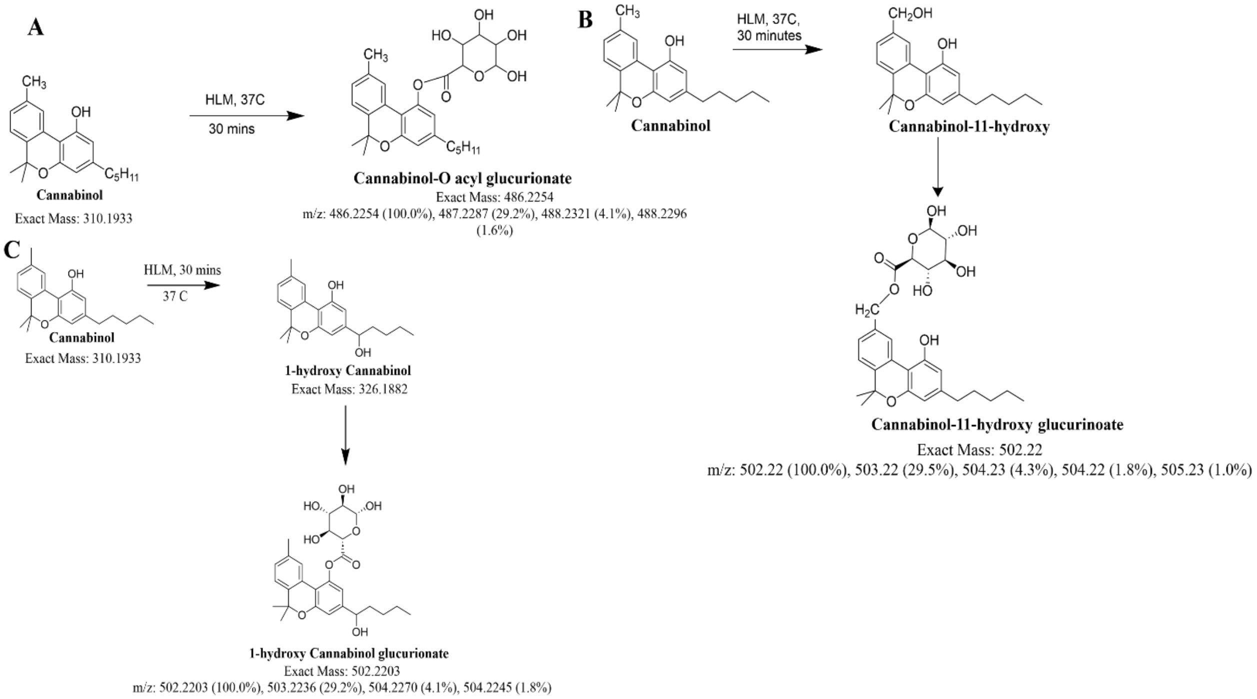

Figure S2 (A) CBN metabolism by HLM resulting the formation of CBN glucurionate **(B)** CBN-11-OHCBN-1'-OH and its glucuronidated product, and **(C)** CBN-1'-OH and its glucuronidated product

Figure S3: LC/MS of CBN and CBN-11-OH glucuronidation

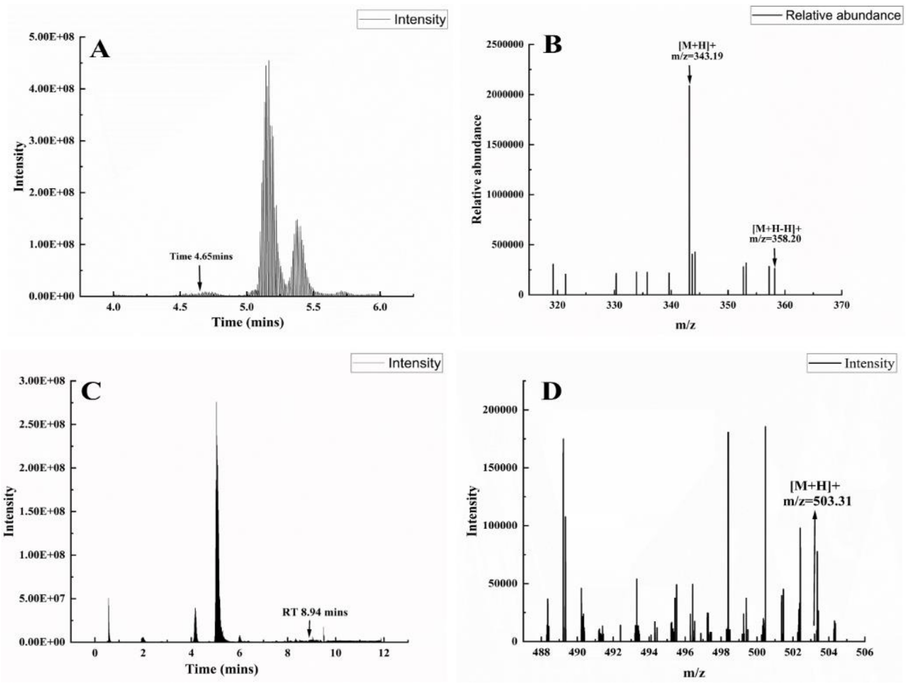

Figure S3: LC and MS1 analysis of CBN-11-OH by Human Liver microsomes **(A)** The hydroxylated CBN-11-OH is detected at 4.65 minutes when subjected to metabolism by HLM, and the respective MS1 showing both single and double hydroxylated m/z in **(B).** The glucuronidated CBN-11-OH peak is detected at 8.94 minutes in **(C)** upon metabolism by HLM, and the respective MS1 shows the m/z of CBN-11-OH-gluc in **(D)**

2.0- Discussions on the binding of CBN and other substrates with CYP2D6, CYP3A4

Nanodisc binding experiments with CBN

We used the CYP3A4 incorporated into the nanodisc for binding experiments with Cannabinol (CBN), Bromocriptine (BCT) and CBN-bound BCT to assess competitive binding between BCT and CBN. The concentration range for Cannabinol for the binding experiment was 0-50µM. We performed the experiment thrice independently and calculated the Km and Vmax using the Michaelis-Menten equation. The Km obtained for CBN binding with CYP3A4 in nanodisc was 12.83733±8.124119 µM. The increasing concentrations of CBN induced a Soret shift at the 417nm and induced a type I shift. We also performed a similar binding experiment with Bromocriptine. The concentration range for Bromocriptine we used was between 0-30 µM. Bromocriptine bound

with CYP3A4 in a much tighter fashion, as indicated by the lower Km value of 1.334±0.292 µM. In order to further assess any sort of binding of CBN with CYP3A4 in nanodisc, we also attempted the binding of Bromocriptine in the presence of 40µM CBN. The Km of bromocriptine in the presence of CBN obtained was 1.381±0.452 µM. The presence of CBN does not seem to significantly alter the binding affinity of Bromocriptine with CYP3A4, indicating that CBN does not have a strong binding affinity for CYP3A4 (Figure S4).

Figure S4: Binding of Cannabinol and Bromocriptine with CYP3A4 in nanodisc

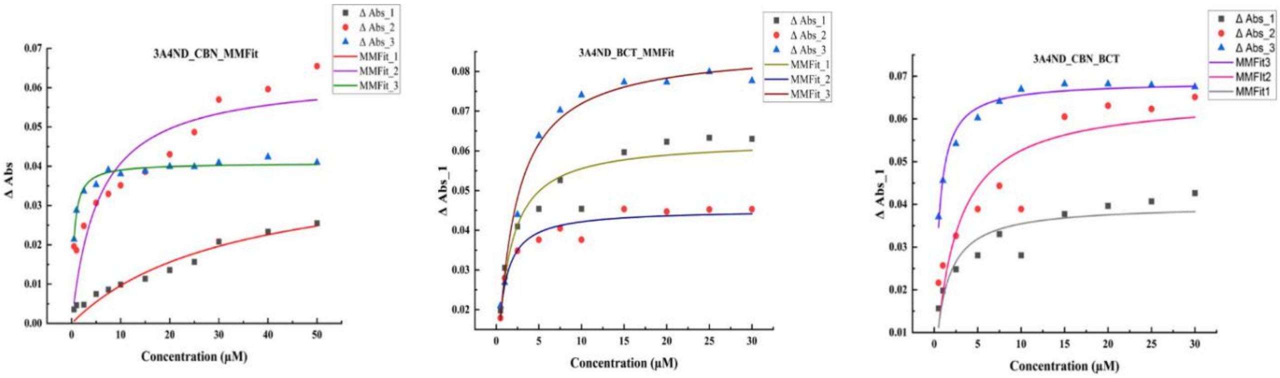

Fig S4: Binding of Cannabinol, Bromocriptine and Bromocriptine in the presence of Cannabinol with CYP3A4 in nanodisc.

2.1-NADPH Oxidation Assays

We also explored the NADPH oxidation rates in the presence of both CBN and Bromocriptine. We observed that in the presence of CBN, the rate of NADPH oxidation slightly decreases as compared to the rate without CBN. The rate of NADPH consumption without CBN (CYP3A4+CPR) was 258.316±19.252 nmol/min/nmol, whereas in the presence of CBN was 249.731±14625 nmol/min/nmol. In the case of Bromocriptine, the rates obtained with and without BCT were 60.788±19.569 nmol/min/nmol and 64.5975±13.671 nmol/min/nmol. We did the experiment in triplicate (Figure S5).

Figure S5: NADPH Oxidation of CBN and BCT with CYP3A4 ND

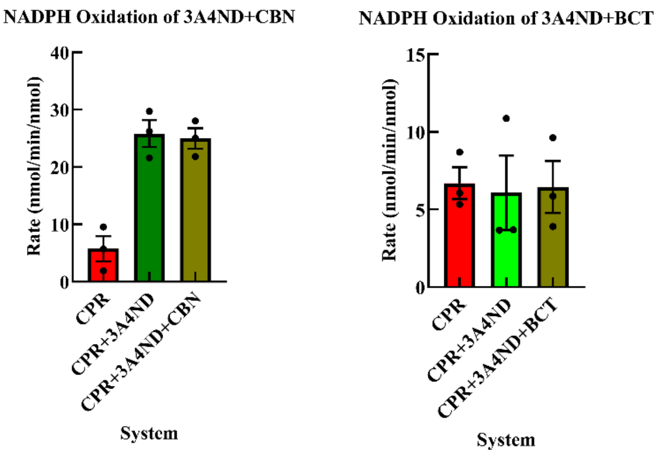

Fig S5: NADPH Oxidation assays with Cannabinol and Bromocriptine.

2.2- CYP2D6 Nanodisc binding experiments with CBN

We used the CYP2D6 incorporated into the nanodisc for binding experiments with Cannabinol. The concentration range for Cannabinol for the binding experiment was 1-80µM. We performed the experiment thrice independently and calculated the Km and Vmax using the Michaelis-Menten equation. The Km obtained for CBN binding with CYP2D6 in nanodisc was 20.656±10.151 µM. The increasing concentrations of CBN induced a Soret shift in the 417 nm and induced a type I shift. The absorbance and the difference spectra are represented in Figure S6.

Figure S6: Binding of CBN with CYP2D6 in nanodisc

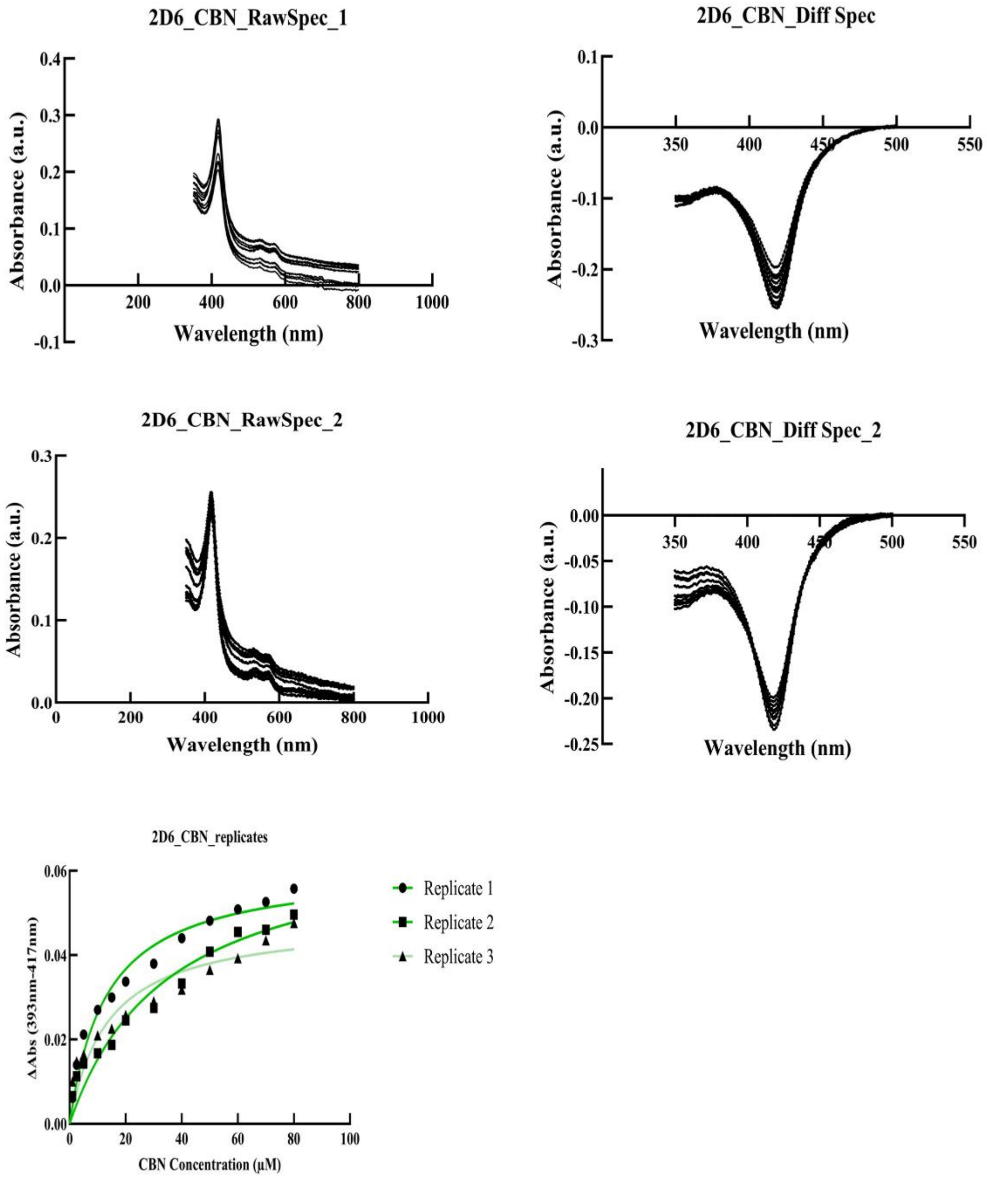

Figure S6: Absorbance Spectra and Difference spectra of CBN binding with CYP2D6 in nanodisc. The pair of absorbance and difference spectra is from one replicate. The experiments were repeated for n=3 independently.

Figure S7: Effect of CBN and its metabolites (LDH and Arginase Assay)

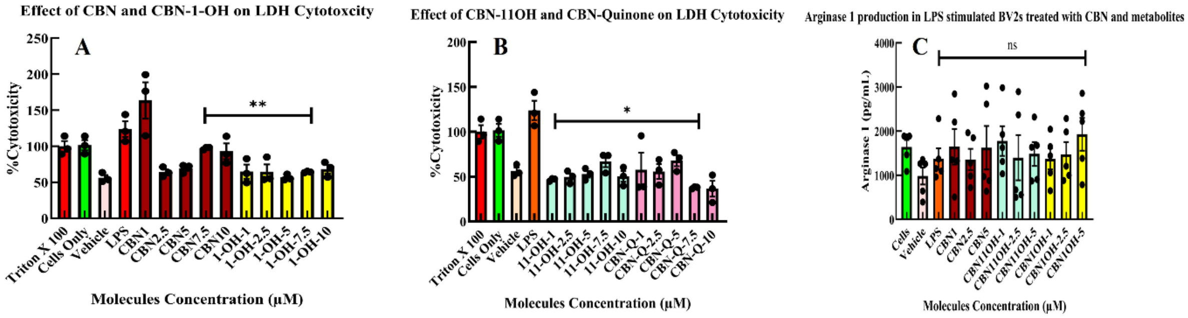

Figure S7: **(A)** Effect of CBN and CBN-1’-OH LPS-induced LDH cytotoxicity **(B)** Effect of CBN-11-OH and CBN-quinone on LPS-induced LDH cytotoxicity and **(C)** Arginase 1 levels measured through Arginase 1 ELISA in LPS-stimulated BV2 cells treated by CBN and its hydroxy metabolites across different concentrations.

.

Figure S8: *In silico* work on THC binding with CYP2C9

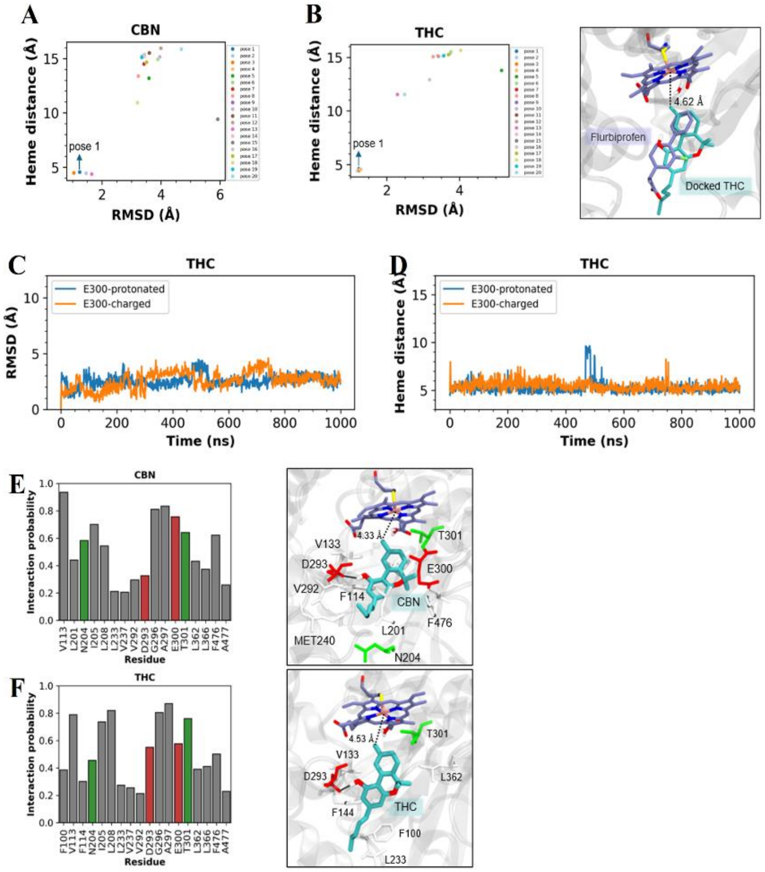

Figure S8: Modelling of CBN and THC binding to CYP2C9 active site using docking and MD simulations (A-B). **The two selection criteria for the docked poses for CBN and THC.** The "Heme distance" refers to the distance between the carbon atom(s) that receive the hydroxy group in each cannabinoid (CBN-C11 and THC-C11) and the Fe atom. The "RMSD" represents the distance between the COM of the flurbiprofen rings and the COM of the ring systems of each cannabinoid. The selected docked pose for each cannabinoid used in the MD simulations is highlighted with an arrow and labeled on the plot. **(B)** The selected docked pose of THC for MD simulations is shown in cyan (carbon), with the crystal ligand, flurbiprofen, depicted in lavender (carbon). The distance between THC-C11 and the Fe atom (pink) is represented by black dashed lines. Fluorine, oxygen, sulfur, nitrogen, and hydrogen atoms are colored green, red, yellow, blue, and white, respectively. C418 and the water molecule coordinating the heme group are explicitly depicted. **(C)** Heavy atom RMSD of THC relative to its initial docked pose during the MD simulations. **(D)** The distance between the THC-C11 and the heme group during the MD simulations. (C-D) Results are presented for two CYP2C9 models: one with protonated E300 (blue) and one with charged E300 (orange). **(E-F)** CYP2C9 residues interacting with cannabinoids. On the left, the residues that interact with each cannabinoid for more than 20% of the MD simulation are shown. Acidic, basic, polar, and hydrophobic residues are represented in red, blue, green, and gray, respectively. On the right, representative snapshots from the MD simulations highlight the residues within 4 angstroms of each cannabinoid (carbon shown in cyan). The hydrogen bond between D293 and cannabinoids is depicted as a black dashed line, and the distance between the selected carbon atom(s) of each cannabinoid (CBN-C11 and THC-C11) and the Fe atom (pink) is also represented by a black dashed line. Oxygen, sulfur, nitrogen, and hydrogen atoms are colored red, yellow, blue, and white, respectively. C418 and the water molecule coordinating the heme group are explicitly depicted. Results are presented for the CYP2C9 model with charged E300.

Figure S9: LC/MS/MS of CBN and CBN-11-OH standard molecules

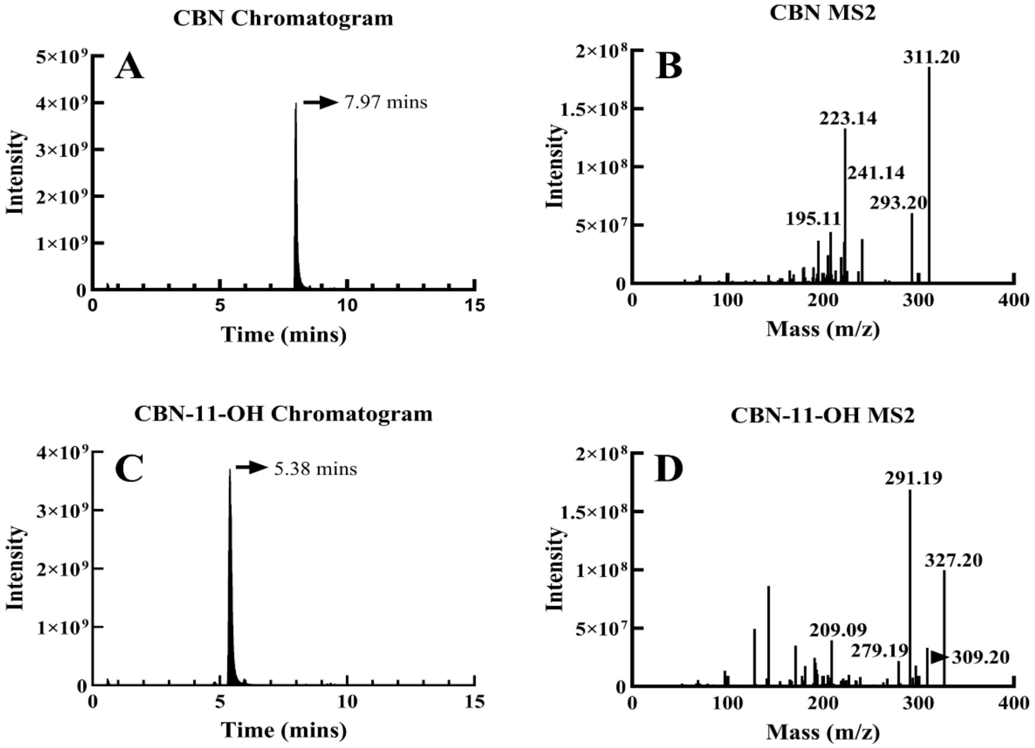

Figure S9: Standard plots of (A) and (C) LC of Cannabinol (CBN) and CBN-11-OH and (B) and (D) of MS2 of CBN and CBN-11-OH

Table S1: Docking score of the best selected pose for CBN and THC

Cannabinoid	Docking score of the best pose (kcal/mol)	Docking score of the selected pose for MD simulation (kcal/mol)	Average docking score ± Standard Deviation (kcal/mol)
CBN	-7.60 (pose 1)	-7.60 (pose 1)	-6.97 ± 0.22
THC	-7.72 (pose 1)	-7.72 (pose 1)	-7.30 ± 0.24

Table S2: Cannabinol and THC showing H-bond presence with D293 residue

**Cannabinoid**	**Residue**	**H bond-presence**
CBN	ASP293	18.50%
THC	ASP293	26.00%

'
